# FlowMat: a toolbox for modeling flow reactors using physics-based and machine learning approaches for modular simulation, parameter identification, and reactor optimization

**DOI:** 10.1039/d5ra06173c

**Published:** 2025-09-12

**Authors:** Sebastian Knoll, Klara Silber, Jason D. Williams, Peter Sagmeister, Christopher A. Hone, C. Oliver Kappe, Martin Steinberger, Martin Horn

**Affiliations:** a Institute of Automation and Control, Graz University of Technology Inffeldgasse 21b 8010 Graz Austria martin.horn@tugraz.at; b Center for Continuous Synthesis and Processing (CCFLOW), Research Center Pharmaceutical Engineering GmbH (RCPE) Inffeldgasse 13 8010 Graz Austria christopher.hone@rcpe.at; c Institute of Chemistry, University of Graz, NAWI Graz Heinrichstrasse 28 8010 Graz Austria

## Abstract

This paper introduces a versatile, open-source MATLAB/Simulink toolbox for modeling and optimizing flow reactors. The toolbox features a modular architecture and an intuitive drag-and-drop interface, supporting a range of different modeling approaches, including physics-based, data-driven, and hybrid models such as physics-informed neural networks. We detail the toolbox's implementation and demonstrate its capabilities through real-world applications, including the simulation of flow reactors, identification of reaction parameters using experimental data (*e.g.*, transient data), and optimization of reactor operating points and configurations. Experimental validations illustrate the practical applicability and effectiveness of the toolbox, making it a valuable resource for researchers and engineers in the field with the potential of reducing the cost and time required for parameter determination and reactor optimization.

## Abstract 2

This paper introduces FlowMat, a versatile, open-source MATLAB/Simulink toolbox for modeling and optimizing flow reactors. The toolbox supports a modular setup and integrates simulation, parameter identification, and reactor optimization.

## Graphical abstract

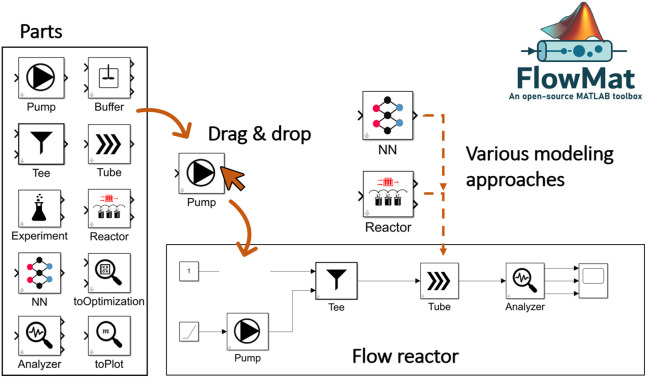

## 1 Introduction

The field of flow chemistry has seen significant advancements in recent years due to the growing need for efficient, scalable, and sustainable chemical processes.^1–3^ Flow reactors offer enhanced control over reaction parameters, improved safety, and the ability to automate processes, making them an essential tool in modern chemistry.^4^ Recent studies highlight the role of flow chemistry in enabling precise control over reactivity and selectivity, which is difficult to achieve in traditional batch processes.^5^ In flow chemistry, the possibility of using transient (dynamic) flow measurements instead of steady-state measurements further increases its applicability. Steady-state measurements involve maintaining constant reaction conditions over time, allowing for the collection of data once the system has reached equilibrium. This approach provides highly accurate and reproducible data but is time-consuming and resource-intensive. Transient flow measurements, on the other hand, capture data during changes in reaction conditions, such as variations in flow rate or temperature. When paired with proper process models, transient measurements can be more efficient and provide insights into the system's dynamic behavior. However, they may be less precise due to the continuous changes in operating conditions when using inappropriate models.

The increased interest in the usage of transient measurements in flow reactors can also be seen in literature. Moore *et al.* presented a method using inline IR spectroscopy and an automated microreactor system to generate time-series reaction data from flow reactors, providing a continuous and efficient alternative to traditional batch experiments for studying reaction kinetics.^6^ Schrecker *et al.* used transient flow methodology to study the Knorr pyrazole synthesis under pH-neutral conditions, uncovering a new intermediate and revising the reaction mechanism, with insights into autocatalysis.^7^ Williams *et al.* reviewed the use of dynamic flow experiments in optimizing chemical processes, emphasizing their role in generating data for accurate kinetic models and identifying optimal conditions for more efficient, sustainable manufacturing.^8^ Aroh *et al.* presented an improved method for reaction kinetics studies using continuous flow microreactors, where simultaneous variation of temperature and flow rate enables rapid concentration profile generation and efficient kinetic analysis.^9^

Moreover, the integration of continuous flow and automation technologies has been shown to significantly enhance efficiency and safety in organic synthesis.^10^ State-of-the-art automation technologies, such as machine learning-driven optimization^11,12^ and robotic platforms,^13^ have enabled precise reaction monitoring and real-time adjustments.^14^ This advance is minimizing human intervention while maximizing efficiency.^15^ Furthermore, recent efforts in autonomous model-based experimental design underscore the potential of combining automation with predictive modeling to accelerate reaction development and significantly reduce the time required for process optimization.^16^

In parallel, modeling approaches have significantly advanced to support the design and optimization of flow systems. Traditional empirical models are now augmented by computational tools that integrate both physics-based models (PBMs) and data-driven models (DDMs).^17,18^ PBMs, such as the tanks-in-series model, and the axial-dispersion model, provide valuable insights into complex phenomena like mixing, heat transfer, and reaction kinetics. The tanks-in-series and axial-dispersion models are widely used to model fluid mixing and dispersion in flow reactors, offering critical insights into residence time distribution and reaction efficiency.^19–21^ Moreover, transfer functions are employed to analyze and predict the dynamic response of flow systems to various input changes, such as variations in flow rate or temperature, enabling precise control and optimization of reactor performance.^22^ In addition, DDM, such as machine learning algorithms, are increasingly utilized to predict system behavior by identifying patterns and relationships within large datasets, allowing for rapid and accurate predictions and optimizations even in complex systems.^23^ These models excel in areas where traditional PBM may struggle, such as handling non-linearities or high-dimensional parameter spaces. Furthermore, emerging hybrid models, such as physics-informed neural networks, combine the strengths of PBM and DDM, leveraging physical laws to guide learning processes and ensuring predictions adhere to known system constraints while benefiting from the flexibility of data-driven approaches.^24,25^

Despite these advances, automation and advanced modeling of flow chemistry remain a challenge.^26^ Specifically, the integration of automation and modeling into flow chemistry continues to face difficulties, particularly in areas such as process control, reaction optimization, process monitoring, scale-up, and data integration.^27^ Efforts are being made to develop user-friendly toolboxes that integrate data acquisition, analysis, and modeling into a single platform for greater convenience. Existing software such as CHEMCAD,^28^ Aspen Plus,^29^ gPROMS,^30^ and Python-based libraries such as OpenFOAM^31^ or Cantera^32^ provide valuable resources for simulation and optimization. While they are powerful for simulation and optimization, they often can be complex and require significant expertise to operate effectively, limiting their accessibility for non-expert users. Furthermore, while Python-based libraries including OpenFOAM and Cantera are flexible and open-source, they can be challenging to configure and may require substantial computational resources for large-scale simulations. Additionally, some of the commercial solutions, such as CHEMCAD, Aspen Plus, and gProms, are costly, making them less accessible for smaller organizations or academic researchers.

**Fig. 1:**
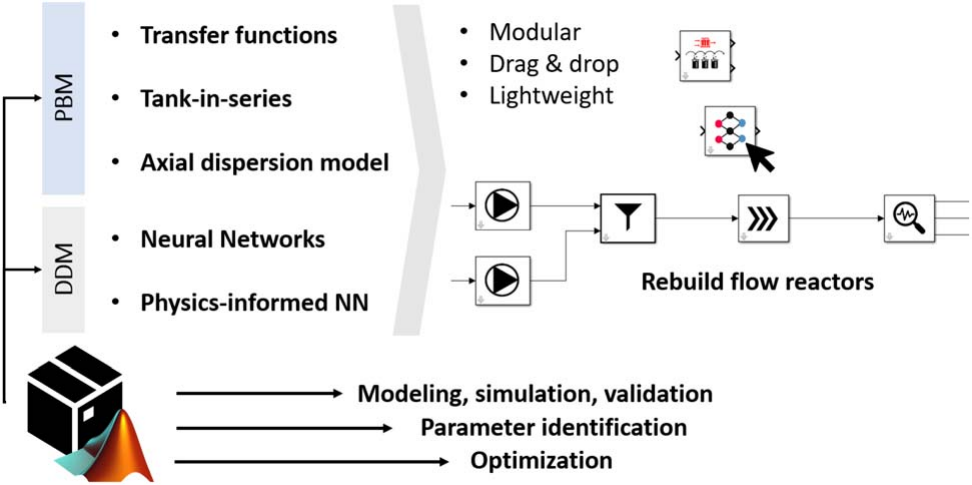
Concept of our developed toolbox highlighting the various modeling approaches, the simple drag and drop system to build flow reactors and the options of simulation, parameter identification, and optimization.

Thus, we present a lightweight MATLAB/Simulink toolbox for modeling flow reactors in a modular way. We intentionally chose MATLAB due to its widespread use in academia and industry, especially in pharmaceutical and chemical engineering. Many users are already familiar with MATLAB, enabling faster adoption, easy integration into workflows, and connection to lab equipment. Our toolbox targets small-scale continuous flow applications with user-friendly, transparent modeling including physics-informed neural networks which are not commonly supported by commercial tools. Importantly, it offers the opportunity for users to deepen their understanding and skills through transparent model structures and detailed documentation. Our toolbox supports various approaches like PBM, DDM, and combinations. It includes models such as transfer functions, tanks-in-series models, axial-dispersion models, and neural network-based methods, such as physics-informed neural networks. The toolbox requires minimal input data but allows for detailed model parameter specifications. Users can combine approaches to simulate real experiments, rebuild real-world reactor setups and optimize for reaction parameters. Moreover, it is possible to apply transient experimental data to identify reaction parameters which can reduce time and cost. Additionally, the toolbox aids in optimizing reactor operations and setups and can be used to find the pareto front for a defined setting. In the toolbox, we combine all common modeling approaches into one lightweight solution, allowing users to focus on the analysis and research rather than the implementation of models or optimizations. An overview of the concept of the developed toolbox can be found in Fig. 1.

The paper is organized as follows. First, we cover the necessary theoretical background and notations of the modeling approaches. Next, we introduce the toolbox, detailing the implementation for each modeling approach and demonstrating how it can be used to simulate real-world reactor setups. In the subsequent section, we describe how reaction parameters can be identified using our toolbox, including details on training a physics-informed neural network to obtain these parameters, and we validate the found parameters using additional experiments. Finally, we show how the toolbox can be used to optimize operating points and the reactor setup itself.

## 2 Theoretical background

In this section we give a small introduction to the necessary theoretical background of all the modeling approaches which we have implemented in our toolbox. We introduce common notations which we will use in subsequent sections.

### 2.1 Transfer function

As shown in literature, transfer functions can be used to describe the input–output behavior of linear time-invariant systems.^22^ When modeling the concentrations of species flowing through a pipe, the resulting delay and dispersion effects can be described using a transfer function. This type of transport-induced delay and dispersion is not equivalent to axial dispersion, which is discussed separately in a later section. Generally, the transfer function can be composed of multiple transfer functions, with parameters and structure adjusted to represent the desired behavior. A common approach combines a dead-time element with a low-pass filter of *n*-th order.

The dead-time element accounts for the nominal delay experienced by species as they flow through the pipe. This delay is given by *τ*_(*L*,*q*)_ = *L*/*q* where *L* denotes the length of the tube, *t* the time, and *q*(*t*) the flow rate of the species. For ideal plug flow, where the species experiences only a delay without any change in the shape of the inflowing concentration, a dead-time element alone would suffice. However, in most cases, the flow through the reactor is not ideal, and additional effects such as dispersion alter the shape of the inflowing concentration. To account for these changes, a low-pass filter of *n*-th order is introduced. The parameters of this low-pass filter can be tuned to accurately describe the observed behavior, including dispersion-induced modifications to the concentration profile of the species.

A corresponding transfer function *H*^*L*,*q*^(*s*) for describing the delay and dispersion effects of an inflowing species with concentration *C̃*^(in)^(*s*) into a tube of length *L* and a constant flow rate *q*(*t*) = *q* can thus be stated as1
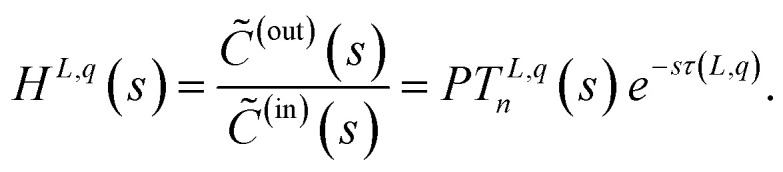


In the equation, *s* denotes the Laplace variable, *C̃*^(out)^(*s*) the outflowing concentration, and the term *PT*^*L*,*q*^_*n*_(*s*) represents the low-pass filter of *n*-th order. The superscript in *H*^*L*,*q*^(*s*) and *PT*^*L*,*q*^_*n*_(*s*) specifies the used tube length *L* and flow rate *q*. As the transfer function *H*^*L*,*q*^(*s*), especially the low-pass filter *PT*^*L*,*q*^_*n*_(*s*), can change for different tube lengths *L* and flow rates *q* it might be necessary, to have separate transfer functions for the individual combinations.

For changing parameters, it is also possible to form the resulting parameters of the transfer function by interpolating the parameters of the transfer function within two (or more) given anchor points. An anchor point, in this context, defines the parameters of the transfer function corresponding to a specific flow rate *q* and length *L*.

### 2.2 Tanks-in-series model

The tanks-in-series model is commonly used in chemical and biochemical engineering to simulate the flow and mixing of substances.^33^ In the tanks-in-series model, the reactor is conceptualized as a series of interconnected tanks. Each tank represents a small compartment of the reactor and it is assumed that each tank is perfectly mixed. Thus, the output concentration of a tank equals the internal concentration. The arrangement allows for the simulation of gradual changes, such as the dispersion of a species or the reaction of a substance as it progresses through the reactor.

**Fig. 2:**
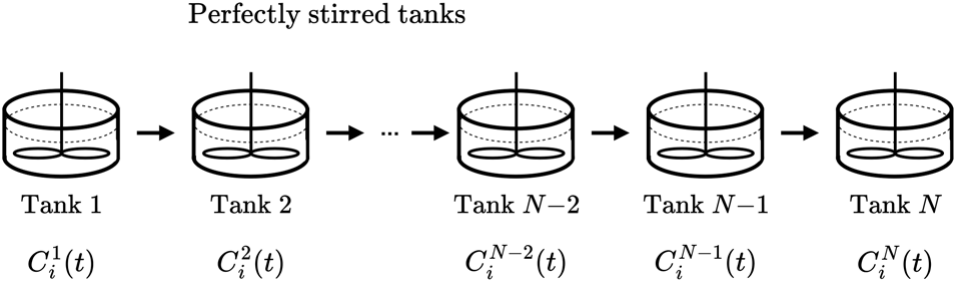
A schematic overview of a tanks-in-series model where the reactor is split into *N* tanks which are perfectly mixed and species are flowing from one tank to the next. (Reproduced from ref. 18 with permission from the Royal Society of Chemistry.)

In Fig. 2, a schematic representation of a tanks-in-series model is shown. The figure illustrates a sequence of *N* interconnected tanks. Species flow sequentially from the first tank to the second, and continuing through the series. The concentration of the *i*-th species in the *j*-th tank is represented by *C*_*i*_^*j*^(*t*).

The change of the *i*-th concentration within the *j*-th tank is given by2
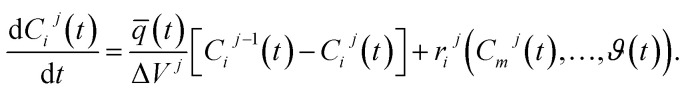


In the equation, Δ*V*^*j*^ denotes the volume of the *j*-th tank, while *q̄*(*t*) represents the volumetric flow rate of the fluid passing through the system. In general, each tank of the tanks-in-series model will be of the same size and thus all tanks will have the same volume Δ*V* resulting in Δ*V*^*j*^ = Δ*V*, ∀*j* ∈ {1, 2, …, *N*}. The term *r*_*i*_^*j*^(*C*_*m*_^*j*^(*t*),…,*ϑ*(*t*)) accounts for changes due to reactions. The underlying reactions can depend on various factors, including other concentrations within the current tank *j* or the temperature *ϑ*(*t*). As the reactors are usually heated evenly across their entire length, it is assumed, that the temperature *ϑ*(*t*) remains uniform throughout the entire reactor system. The input to the tanks-in-series model consists of all the inflowing concentrations *C*^(in)^_*i*_(*t*). As these concentrations enter the first tank, the relationship *C*^0^_*i*_(*t*) = *C*^(in)^_*i*_(*t*) applies. The number of tanks *N* can be used to simulate various degrees of mixing within the tanks-in-series model. When choosing *N* = 1 the tanks-in-series model represents a homogeneously mixed reactor while for *N* → ∞ ideal plug flow is modeled.

### 2.3 Axial-dispersion model

The axial-dispersion model is commonly used in chemical engineering to describe the behavior of flow and mixing in tubular reactors or packed beds. It accounts for deviations from ideal plug flow by introducing a dispersion term that represents the spreading of species along the axial direction of the system. The axial-dispersion model bridges the gap between plug flow and complete mixing by incorporating the effects of molecular diffusion and flow irregularities. The key parameter in the model is the axial-dispersion coefficient *D*, which quantifies the extent of mixing along the reactor axial direction *z*. This form of axial dispersion differs fundamentally from the delay-dispersion effects discussed earlier in the context of transfer function modeling. While the former captures physical mixing effects caused by molecular diffusion and flow heterogeneities along the reactor axis, the latter typically models transport behavior in a more abstract way using dynamic system elements such as dead-time and low-pass filters.

**Fig. 3:**
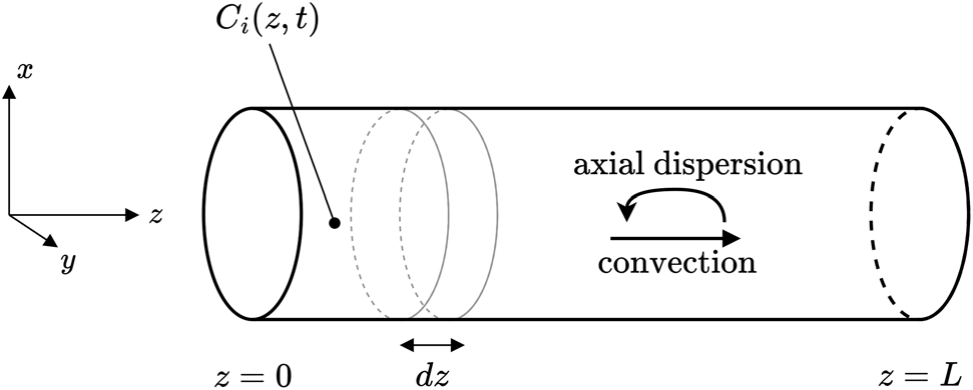
A schematic overview of the effects considered within the axial-dispersion model.

In Fig. 3 a reactor of length *L* with the main effects of the axial-dispersion model is depicted. One can see, that the concentration *C*_*i*_(*z*,*t*) of the *i*-th species is given as function of space *z* and time *t*. Moreover, not only convection due to the flow rate *q*(*t*) is present but also axial-dispersion effects.

The axial-dispersion model is given by the partial differential equation (PDE) of the form3
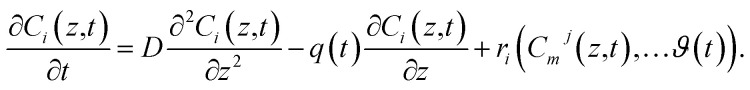


In the model, *C*_*i*_(*z*,*t*) denotes the concentration of the *i*-th species, *D* denotes the axial-dispersion coefficient and *q*(*t*) the flow rate of the solute through the system. Reactions are accounted for by the term *r*_*i*_(*C*_*m*_^*j*^(*z*,*t*),…,*ϑ*(*t*)), which can depend on various factors, including other concentrations, and the temperature *ϑ*(*t*).

### 2.4 Neural network

Neural networks are models which are inspired by the structure and functioning of the human brain. A neural network consists of interconnected nodes, also known as “*neurons*” which are organized into layers. The neural network processes input data through weighted connections and applies activation functions to generate outputs. This mechanism allows them to recognize patterns, make predictions, or classify information.

**Fig. 4:**
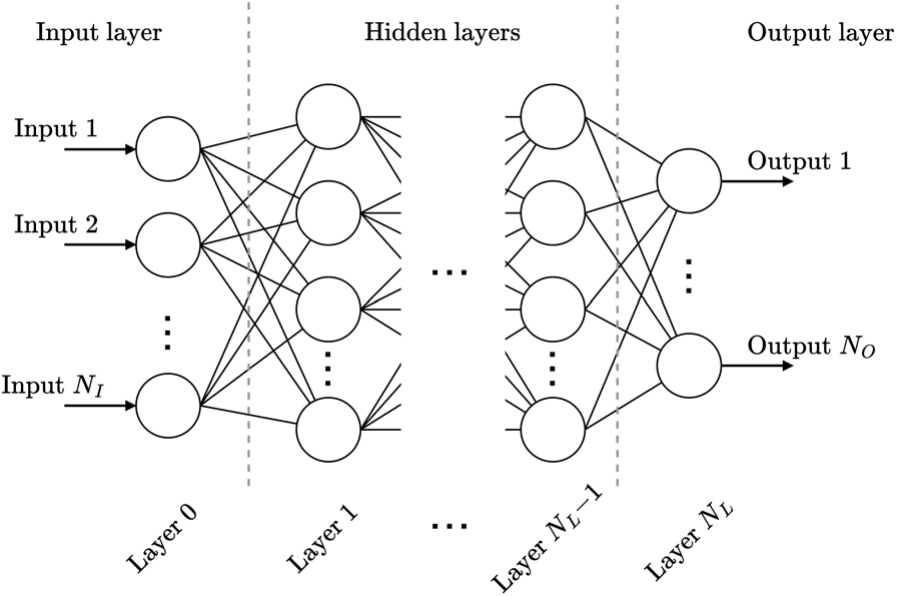
A schematic overview of the architecture of a fully connected neural network.

In Fig. 4, a fully connected multi-layer perceptron neural network is illustrated. As shown, the neural network is structured into three main components: an input layer, one or more hidden layers, and an output layer. Each layer comprises a specific number of neurons, in which every neuron in a layer is connected to all neurons in the preceding layer. These connections transmit the output values of the previous neuron to the next neurons, whereby each connection has an assigned weight that scales the transmitted value. Within a neuron, the weighted sum of all incoming connections plus a bias is computed, and an activation function is applied to this sum. The activation function, which can be non-linear (*e.g.*, sigmoid or softmax), transforms the value before passing it to the neurons in the next layer. The number of neurons in the input layer must match the number of input features *N*_I_, while the output layer must have as many neurons as there are target labels *N*_O_. The architecture of the hidden layers, including the number of neurons and layers, depends on the complexity of the specific problem. According to the universal approximation theorem,^34,35^ even a shallow neural network with a sufficient number of neurons can approximate any continuous function under certain conditions. This demonstrates the theoretical capability of a neural network for a wide range of tasks.

Given the introduced structure, one can define a neural network 
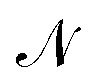
 with *N*_L_ + 1 layers, as4
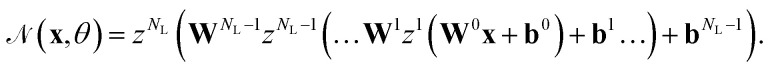


In eqn (4), *θ* represents the neural network parameters consisting of the matrices **W**^*i*^ and the bias vectors **b**^*i*^, ∀*i* ∈ {0, …, *N*_L_ − 1}. Thereby, the matrices **W**^*i*^ and the bias vectors **b**^*i*^ represent the connections of the neurons from layer *i* to the next layer *i* + 1 and the bias values of each neuron within the *i*-th layer respectively. The activation functions within the *i*-th layer are represented by *z*^*i*^.

The goal of the neural network 
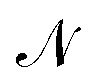
 is to reconstruct an unknown function 
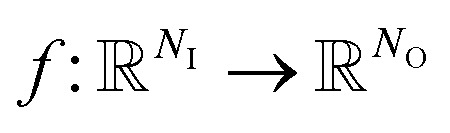
 such that 
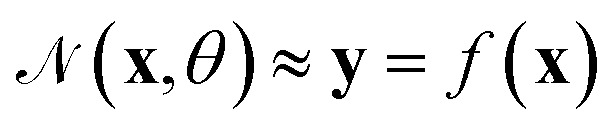
 where 
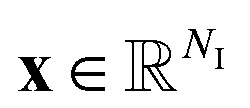
 denotes the input. To achieve that the neural network 
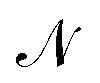
 maps an input 
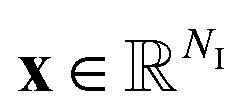
 to the corresponding output 
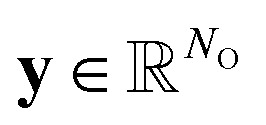
 accordingly, the neural network 
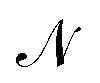
 has to be trained. In the training, the parameters *θ* of the neural network 
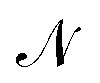
 are optimized in such a way, that a defined loss function 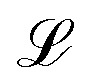 is minimized. Given a set of *N*_S_ input–output samples {(**m**_*k*_, **n**_*k*_): *k* = 1, …, *N*_S_}, where 
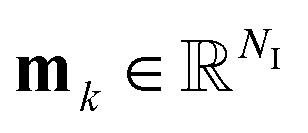
 is a input-sample, 
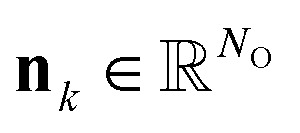
 the corresponding output-sample, and the prediction 
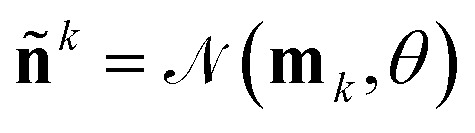
 of the neural network 
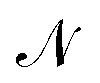
, the loss function can be defined as5
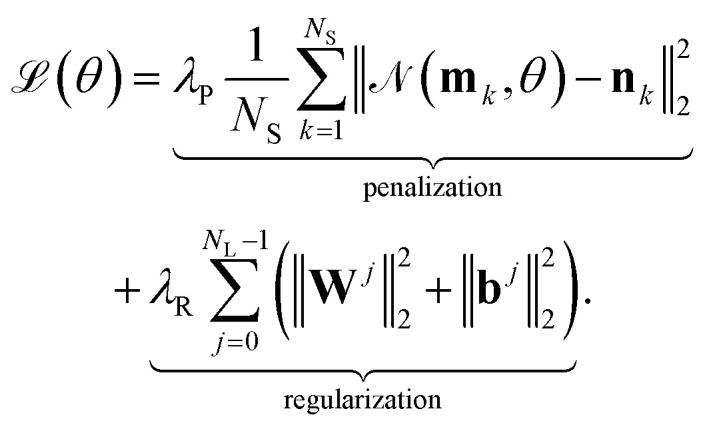


In the equation, ‖·‖_2_ denotes the *L*_2_-norm and one can see, that the loss function 
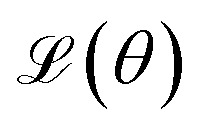
 combines a *penalization* term and a *regularization* term, each weighted by the factors *λ*_P_ and *λ*_R_, respectively. Generally, these terms take various forms: the *penalization* term penalizes deviations of predicted values from the actual values, while the *regularization* term mitigates overfitting by discouraging extreme weights in the neural network 
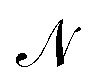
. Minimizing the loss function 
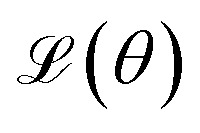
 involves an iterative process, where each iteration consists of a forward and a backward pass. In the forward pass, the loss is computed based on the current neural network parameters *θ*. During the backward pass, the parameters *θ* are updated using a gradient-based method derived from the computed loss. This process is commonly referred to as “*backpropagation*”.

### 2.5 Physics-informed neural network

While data-driven approaches, such as neural networks, have shown great promise in modeling complex systems, they can be impractical for flow chemistry applications. This is primarily because these methods rely heavily on large amounts of experimental data, which can be expensive and time-consuming to collect. Additionally, in many cases, the necessary data may not be available at all. This is where physics-informed approaches, like physics-informed neural networks, offer a significant advantage.

The principle of physics-informed neural networks lies in their ability to integrate physical laws, represented by PDEs or other governing equations, directly into the neural network training process. Unlike traditional neural networks that rely solely on data for training, physics-informed neural networks incorporate these physical principles as part of the loss function. This approach ensures that the learned solution not only fits the available data but also covers the underlying physical laws, such as conservation laws, boundary conditions, or dynamic equations. By embedding this additional layer of information, physics-informed neural networks achieve higher accuracy with smaller datasets and can model complex systems where data might be sparse or noisy, making them particularly powerful for solving scientific and engineering problems.

For the integration of the physical principles into the training of the neural network, the loss function of the physics-informed neural network can be defined as6
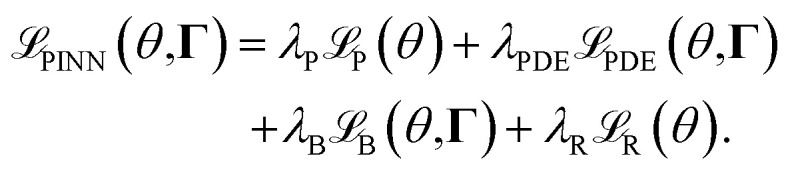


In the equation, 
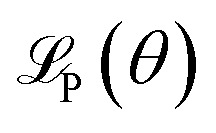
 represents the loss from the *penalization* term, while 
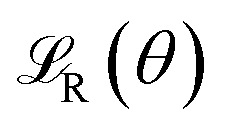
 corresponds to the *regularization* term similar as in the loss of a neural network. The terms 
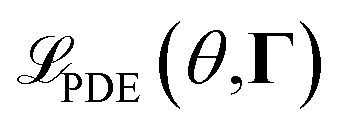
 and 
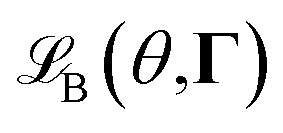
 represent the losses associated with the incorporated physical principles and the boundary conditions, respectively. The two latter terms depend on physical parameters, collectively denoted as **Γ**. These terms are formulated to measure the deviations between the predicted behavior and the physical behavior. Each of the terms is multiplied by a factor *λ* to appropriately weight their contributions relative to one another.

By setting *λ*_PDE_ and *λ*_B_ to zero, the corresponding terms in the loss function vanish, reducing the setup to a standard neural network training configuration without physics-informed constraints. On the other hand, setting *λ*_P_ to zero enables the training of a neural network solely to reconstruct the PDE solution without relying on any data. A balanced combination of these factors allows for a trade-off between utilizing data-driven learning and incorporating physical knowledge into the model. This approach also provides the flexibility to identify the physical parameters **Γ**, ensuring that the model aligns with both empirical observations and the underlying physical principles.

## 3 Results and discussion

In this section, we provide an in-depth overview of the implementation and capabilities of the developed toolbox. We also explain how the toolbox can be used to build flow reactor setups and validate them with real-world setups and experiments. Additionally, we discuss how the toolbox can be used to identify parameters through various methods, explain the validation process for those parameters, and demonstrate its application in optimizing reactor operating points and the reactor setup itself.

### 3.1 The FlowMat toolbox

Given the variety of modeling approaches available, it is often challenging to choose the correct one. Moreover, once a modeling approach is chosen, the implementation and theoretical background can be complex and not straightforward especially for a non-specialist in those techniques, *e.g.* a process chemist. Thus, we present a lightweight open-source MATLAB/Simulink toolbox that combines the most common modeling approaches within one solution. Users can choose between PBM, DDM, or combinations, such as physics-informed neural networks. *Via* drag-and-drop, it is possible to select common reactor parts and rebuild a flow reactor, requiring only the most necessary parameters for each element. No further implementation or detailed mathematical understanding is necessary to use these parts. If more detailed parameters are required, they can also be entered. The FlowMat toolbox can be used to simulate, analyze, and optimize reactor systems. Parameters can be entered from other sources or identified using the toolbox. For the identification of parameters various methods are possible whereby one is to train a physics-informed neural network using experimental data. Here, the real-world reaction and reactor parameters are a byproduct of the training of the physics-informed neural network.

**Fig. 5:**
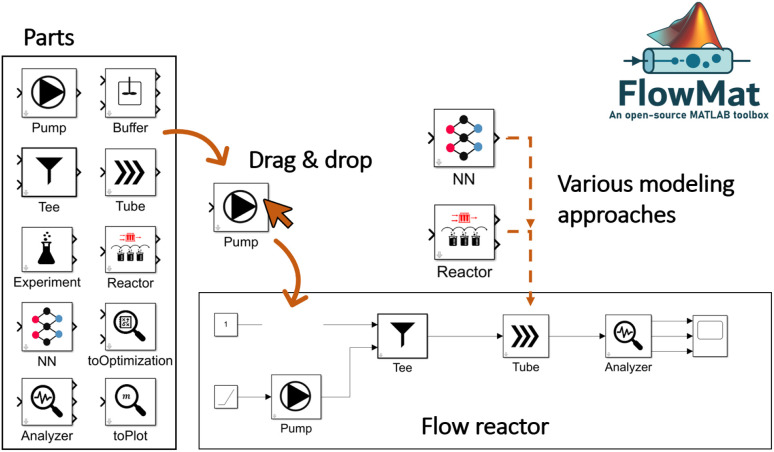
Illustration and concept of the FlowMat toolbox.

In Fig. 5 the concept of the toolbox is depicted. One can see, how a flow reactor is built by choosing the necessary parts out of the toolbox. It is possible to select from various modeling approaches the best option or use them in combination.

### 3.2 Implementation

The implementation was done in MATLAB and Simulink. MATLAB/Simulink is a commercial software which is highly used in educational background and universities. In general, the reactor parts are implemented as individual Simulink blocks to maintain the idea of a modularity. All parts communicate *via* one interface such that each part can be connected with other ones. This interface contains only necessary information such as the flow rates of each species and the corresponding substance concentrations. Along with these Information also the temperature is transferred within the common interface.

Before using the toolbox, the toolbox has to be initialized whereby all the necessary variables and information of the interface are generated. The initialization of the toolbox can be achieved by calling one provided method within a MATLAB/Simulink script.

In addition to implementing the most common modeling approaches, discussed in more detail in the following subsections, we developed various reactor components, including *Pumps*, *Buffers*, *Tees*, *Experiments*, *Analyzers*, *toOptimization*, and *toPlot*. *Pumps*, *Buffers*, and *Tees* are used to model inputs and flow distribution within the system. *Analyzers* enable the analysis of concentrations, flow rates, and temperature at specific points of interest. The *Experiments* block facilitates the incorporation of input data and measured concentrations into the project, allowing for comparison and ensuring consistency with real-world inputs. The *toOptimization* block seamlessly transfers setup information to an optimization task, while the *toPlot* block simplifies the visualization of output data, such as concentrations.

#### 3.2.1 Implementation of a transfer function

The first Simulink-block is using the modeling approach of transfer functions. As described in Section 2.1 we can use transfer functions, to model delay effects and dispersion effects. For the implementation of the according Simulink-block we make use of the introduced form whereby we use a low-pass filter of 2-nd order which in general can be extend to any arbitrary order. The resulting transfer function, for a given tube with length *L* and a given flow rate *q* can be written as
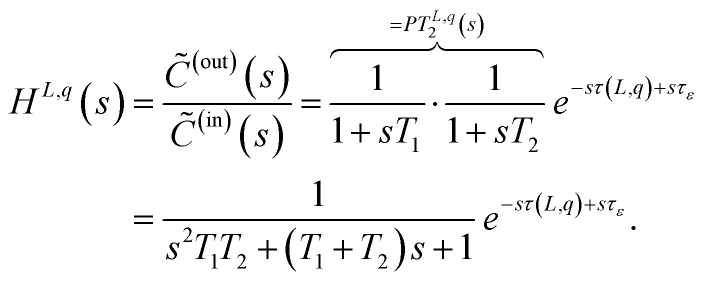


**Fig. 6:**
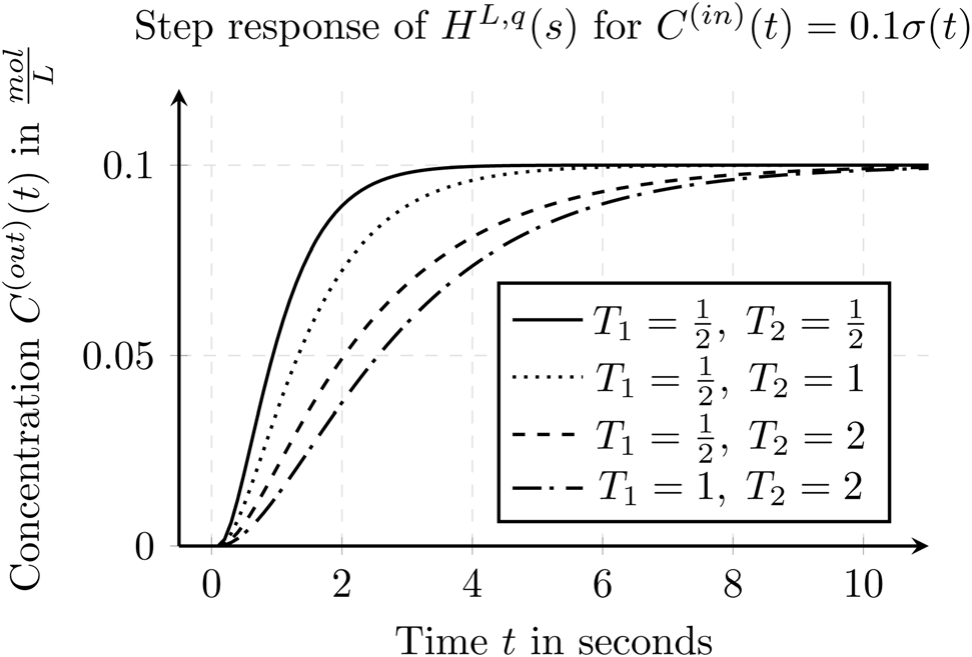
Step response of a transfer function using a low-pass filter of 2-nd order for various time constants *T*_1_ and *T*_2_ and setting the nominal delay *τ*(*q*, *L*) = 0 and *τ*_*ε*_ = 0.

In the equation, the time constants *T*_1_ and *T*_2_ define the effect of the dispersion effects. Additionally, a delay offset *τ*_*ε*_ can be used, if the real-world behavior shows slightly different results within the nominal delay than expected from theory. The resulting dispersion effects for different time constants *T*_1_ and *T*_2_ are depicted in Fig. 6. In the figure, a step from 0 to 0.1 at time *t* = 0 on the inlet concentration is applied. This step is often denoted by *C*^(in)^(*t*) = 0.1*σ*(*t*). In the figure, one can see, that the step becomes smoother and more curved as the time constants *T*_1_ and *T*_2_ increase.

As the dispersion effects might change for different flow rates *q* and tube lengths *L*, also the time constants *T*_1_ and *T*_2_ have to change for the different settings. Thus the Simulink block provides the possibility, to define several anchor points for different flow rates *q* and a given tube length *L*. For flow rates in between two anchor points the parameters *T*_1_, *T*_2_ and the delay offset *τ*_*ε*_ are linearly interpolated to allow a smooth transition and to allow to have flow rates in between two anchor points.

For the implementation in MATLAB/Simulink, the time-continuous transfer function *H*^*L*,*q*^(*s*) is discretized using a definable discretization time *T*_d_ and using the discretization Method of *Tustin*.^36^ Using the Method of *Tustin*, the discretized transfer function *H*^*L*,*q*^_d_(*z*) is found by7
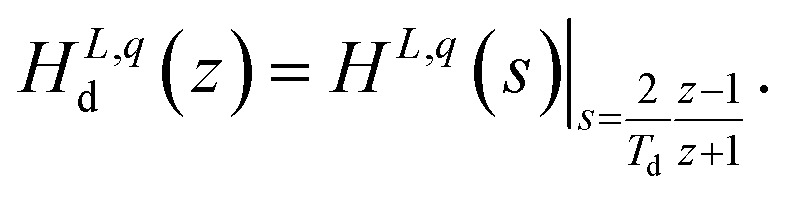


#### 3.2.2 Implementation of the tanks-in-series model

For the implementation of the tanks-in-series model, eqn (2) is used to form a state-space model of the entire tanks-in-series model. The state-space vector **x** is defined to hold all concentrations *C*_*i*_^*j*^(*t*) for each tank *j* and each species *i*. Assuming that there are *P* species flowing through the system, and we split the reactor in *N* tanks, the state-space vector is defined as

8 **x**(*t*) = [*C*_1_^1^(*t*) *C*_1_^2^(*t*)…*C*_1_^*N*^(*t*) … *C*_P_^1^(*t*) … *C*_P_^*N*^(*t*)]^T^.

This results in the state-space model of the form9
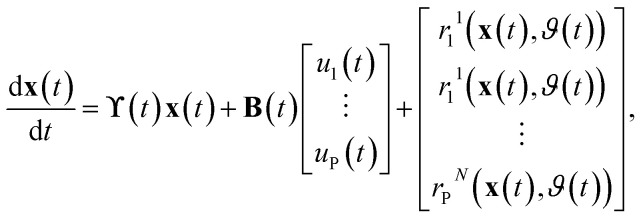
where10
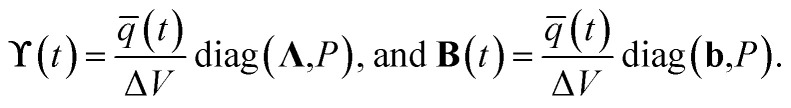


In the equation, the expressions diag(**Λ**,*P*), and diag(**b**,*P*) represent a block diagonal matrix where the matrix **Λ** and the vector **b** are repeated *P* times along the diagonal respectively. As11
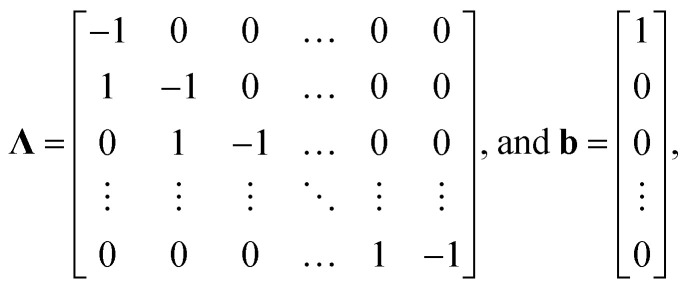
where **Λ** is of size *N* × *N* and **b** of size *N* × 1, we can conclude, that the block diagonal matrix diag(**Λ**,*P*) is of size *NP* × *NP* whereas the block diagonal matrix diag(**b**,*P*) is of size *NP* × *P*. Furthermore, it holds, that *u*_*i*_(*t*) = *C*^(in)^_*i*_(*t*), ∀*i* ∈ {1,…,*P*}. The factors *r*_*i*_^*j*^(**x**(*t*),*ϑ*(*t*)) which consider the reaction term forming species *i* in tank *j* depend on the temperature *ϑ*(*t*), and the state-vector **x**(*t*) as it contains all concentrations. The superscript *j* was added to the factors *r*_*i*_^*j*^ as they now collectively depend on **x**(*t*).

For the implementation, the state-space model is discretized using the ZOH-method^37^ and a definable discretization time *T*_d_. Additionally the implementation allows several reactions within the tanks-in-series model to be defined. The form of the reaction is defined as a second order reaction whereby the reaction rate is described by the Arrhenius equation, which relates the reaction rate to temperature and an activation energy.^38^ Given a reaction where species *m* and species *n* react to species *i*, the reaction within each tank *j* can be stated as12
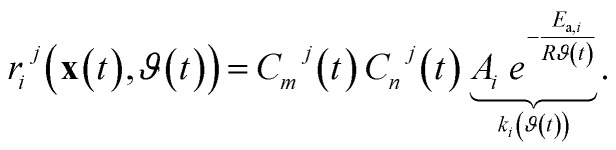


In the equation, *A*_*i*_ denotes the pre-exponential factor, and *E*_a,*i*_ the activation energy of the reaction forming species *i*. The reaction rate *k*_*i*_(*ϑ*(*t*)) is the same throughout all tanks. The temperature in Kelvin is denoted with *ϑ*(*t*) and the universal gas constant with *R*.

#### 3.2.3 Implementation of the axial-dispersion model

For the axial-dispersion model we are facing a PDE of the form stated in eqn (3). We assume, to have a Dirichlet boundary condition at the inlet and a Neumann boundary condition at the outlet of the reactor. The boundary condition for the inlet ensures, that the input concentration *C*^(in)^_*i*_(*t*) is the concentration at the inlet *C*_*i*_(*z* = 0, *t*) of the reactor. The boundary condition for the outlet ensures, that the concentration at the outlet of the reactor *C*_*i*_(*z* = *L*, *t*) cannot change over time, implying a steady-state behavior or negligible transient effects at the reactor's exit. The corresponding boundary conditions ∀*i* ∈ {1, …, *P*} read as13
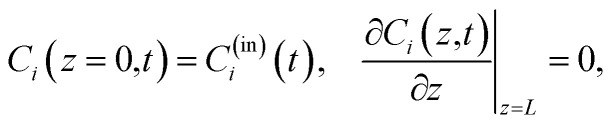
respectively. For the implementation of the axial-dispersion model we first apply a semi-discretization of eqn (3). By applying a discretization within the axial direction *z*, we split the reactor in *N* sub-parts of the same size Δ*z*. Within each sub-part we approximate the derivatives by using the central difference and finite difference method. The resulting equation can be stated as14
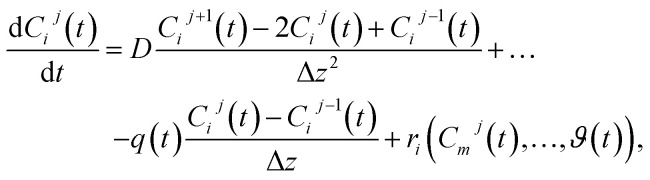
whereby one can see, that we end up with an ordinary differential equation (ODE) within each spatial discretized block *j*. When comparing the resulting form with eqn (2), which is used to describe the tanks-in-series model, and knowing that
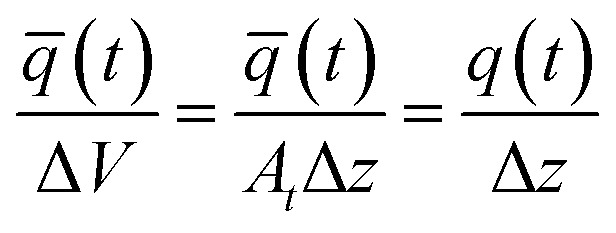
for a constant tube cross section *A*_*t*_, we see, that the equation is the same except the additional term with the axial-dispersion coefficient *D*. We can conclude, that when setting the axial-dispersion coefficient *D* to zero, we will have the same model as for the tanks-in-series model. If the axial-dispersion coefficient *D* is not zero, we have the additional term and we therefore have to adopt **ϒ**(*t*) and **B**(*t*). The resulting state-space model for the axial-dispersion model reads as15
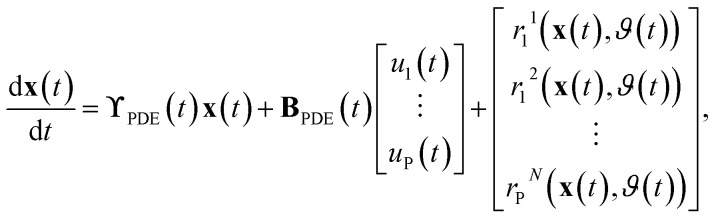
where16
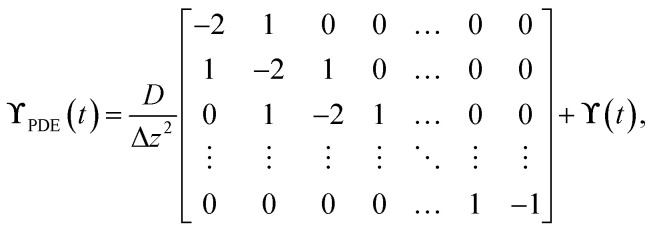
and17
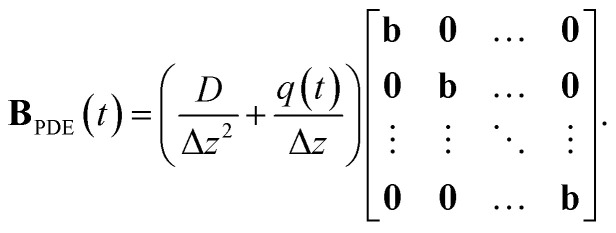


For the implementation, the resulting state-space model is again discretized using the ZOH-method. As the tanks-in-series model and the axial-dispersion model result in a comparable model, both models are implemented within the same Simulink-block. Defining the axial-dispersion coefficient *D* accordingly, one can define which model is used.

#### 3.2.4 Implementation of neural networks

For the implementation of neural networks we make use of the introduced multi-layer perceptron neural networks. The user can define the structure such as the number of hidden-layers and the individual number of neurons. Alternatively the user can use a default structure of a shallow multi-layer perceptron neural network for which the number of neurons is automatically determined based on the number of concentrations *P*. As input vector **x**, the flow rates *q*_*i*_(*t*) for each individual species *i* and the temperature *ϑ*(*t*) are chosen. As the input has to contain discrete values in time, we choose discrete sample times *t* = *kT*_d_ where 
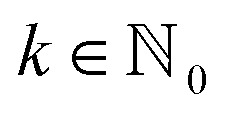
 for a definable sample time *T*_d_. To keep the formulas more readable, we abbreviate quantities evaluated at time *t* = *kT*_d_ using the additional subscript *k*. Moreover, to enhance the estimations of output series, the input vector **x** of the neural network 
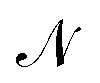
 is extended to also hold previous input values up do definable number *N*_d_. For a given dataset of the input flow rates *q*^(in)^_*i*_(*t*) for all *i* ∈ {1, …, *P*}, the temperature *ϑ*(*t*), and the recorded measurements of the outflowing concentrations *C*^(out)^_*i*_(*t*) the input-output-samples for the training of the neural network 
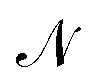
 can be defined as18
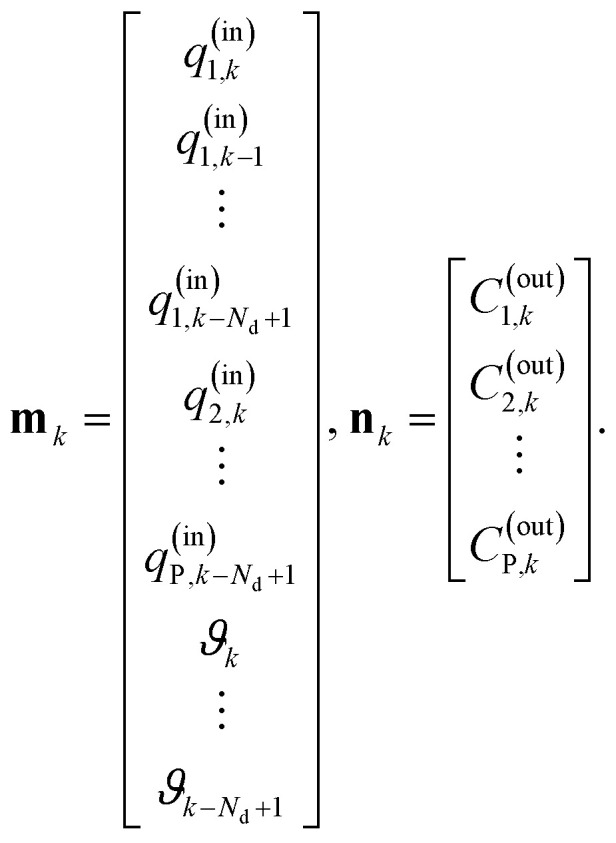


For the training we use the Adam optimization algorithm^39^ to optimize the neural network parameters *θ*. In the implementation, the resulting neural network can be used within a Simulink file or standalone within a MATLAB/Simulink script itself. In general, it is considered good practice to split the available data into separate training and validation (or test) sets. This allows for evaluating the model's ability to generalize and helps prevent overfitting. The toolbox supports this approach by letting users supply only the training data to the training function, while the trained neural network can be evaluated on the remaining validation data. Additionally, the method can be adapted to incorporate test data directly into the training process if needed (for example, for early stopping). Moreover, hyperparameter tuning is commonly applied during neural network training to improve performance. Although no hyperparameter tuning was performed at this stage, the framework is designed to be extensible, enabling users to add such optimization techniques if desired. This could lead to improved model accuracy by systematically selecting the best training configurations.

#### 3.2.5 Implementation of physics-informed neural networks

For the physics-informed neural network we use the same methods to define the structure as for the neural network, whereby we incorporate the axial-dispersion model into the loss function 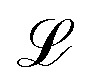 of the neural network. The considered reactions within the axial-dispersion model can be of any form. For instance, one can describe the underlying reaction using the Arrhenius equation. For the integration of the axial-dispersion model, we extend the output **y**_*k*_ of the neural network 
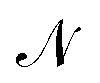
 to include spatial predictions of each concentration by dividing the reactor in *N* + 1 sub-parts. The spatial discretization is comparable to the implementation of the axial-dispersion model in Section 3.2.3. Moreover, we include predictions of physical parameters **Γ** which are required within the incorporated physical equations. When incorporating the axial-dispersion model including reactions, we have to include parameters like the axial-dispersion coefficient *D*, the pre-exponential factors *A*_*i*_ and activation energy *E*_a,*i*_ for each underlying reaction forming species *i*. Assuming, that species 3 and species 4 are formed within a reaction, the lifted output **y**_*k*_ of the neural network 
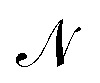
 reads as19
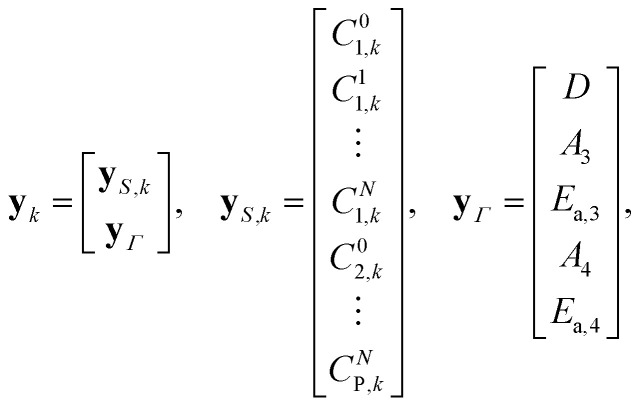
where *C*_*i*,*k*_^*j*^ represents the concentration of the *i*-th species within the *j*-th sub-part at time *t* = *kT*_d_. Since not all concentrations *C*_*i*,*k*_^*j*^ or physical parameters **Γ** can be directly measured, only a subset of the predicted output **y**_*S*,*k*_ can be incorporated into the *penalization* term of the loss function during the training of the physics-informed neural network. Specifically, the *penalization* term can only utilize concentrations that are either measured directly or calculated from flow rates. This includes the input concentrations of the reactor *C*^0^_*i*,*k*_ for all *i* ∈ {1, …, *P*}, which can be measured or derived from the flow rates of individual species, and the output concentrations *C*_p,*k*_^*N*^ for all *p* ∈ {1, …, *G*}. As it may not always be feasible to measure all outflowing concentrations *C*_p,*k*_^*N*^, it holds that *G* ≤ *P*. By combining all measured and known concentrations, we can define the output of a sample as:20
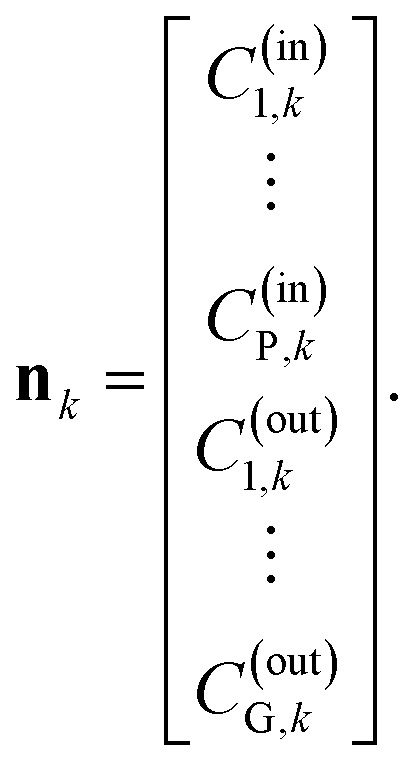


For the input-sample **m**_*k*_ we use the same as for the training of a neural network. When we assume, that we have *N*_s_ input–output samples (**m**_*k*_, **n**_*k*_) for *k* ∈ {1, …, *N*_s_} which can be used for the training, we can define the *penalization* term within our loss function 
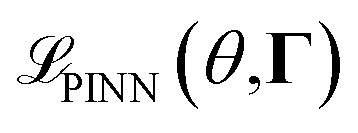
 as21
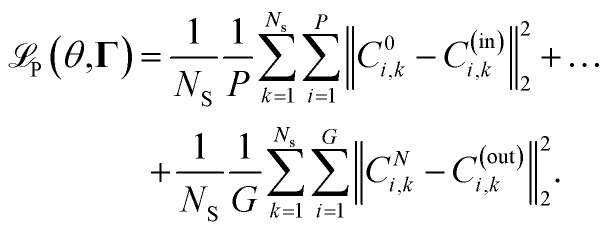


In the equation, the elements *C*^0^_*i*,*k*_, and *C*_*i*,*k*_^*N*^ are extracted from the current predictions of the neural network 
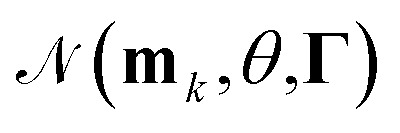
 whereas the elements *C*^(out)^_*i*,*k*_, and *C*^(in)^_*i*,*k*_ are from the output-sample **n**_*k*_.

In addition to the data-driven loss 
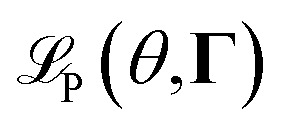
, we can define a cost term derived from the incorporated axial-dispersion model. By discretizing the axial-dispersion model from eqn (14) in both the spatial and temporal dimensions, we can compute the residual and utilize it as a cost factor. The resulting cost term 
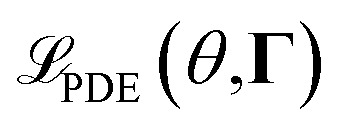
, reflecting the incorporated physical relations, thus reads as:22
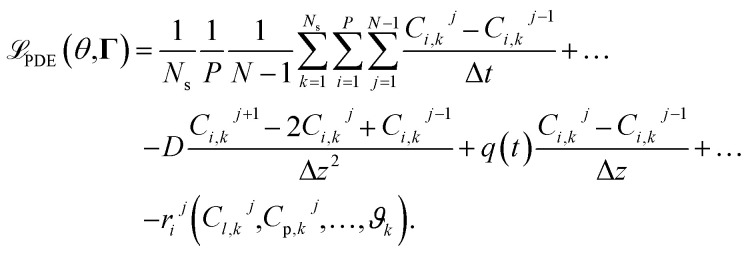


In the cost term, Δ*t* = *T*_d_, 
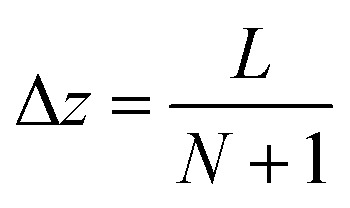
, and each concentration *C*_*i*,*k*_^*j*^ is predicted by the neural network 
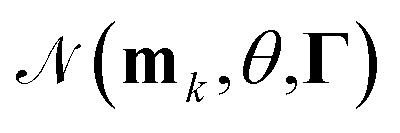
. Additionally, to the two losses and the *regularization* term, which we described in the theoretical background, we add a loss factor to ensure that the physical parameters **Γ** stay within physically feasible bounds. We do that, by defining the additional cost23
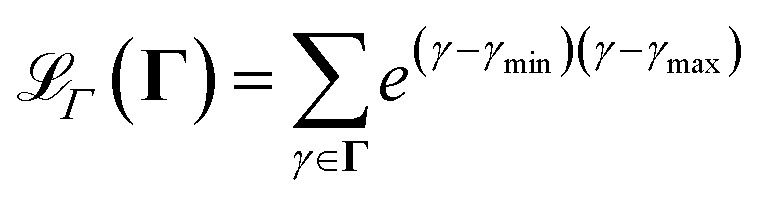
in which *γ* is a physical parameter in **Γ**, and *γ*_min_ and *γ*_max_ are the lower and upper bounds of the feasible values for the physical parameter *γ* respectively.

For the total cost 
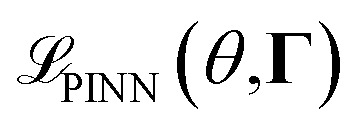
, which is used for the training of the physics-informed neural network, we use the combination of all defined costs and weight them with the terms *λ* relative to each other. The overall cost thus reads as24
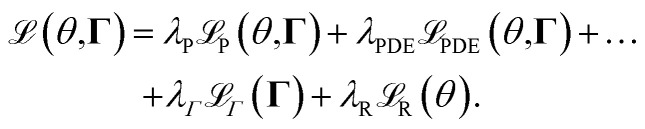


As we penalize any deviations of our boundary conditions (13) already in the penalization term 
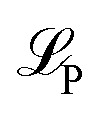
, we can set the cost term due to the boundary conditions 
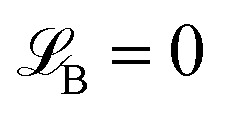
. For the training of the physics-informed neural network 
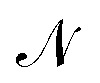
, the Adam optimization algorithm is again employed. As with the neural network training, no hyperparameter tuning was conducted at this stage. However, the method can also be extended to incorporate such optimization for physics-informed neural networks, which may further improve model performance.

### 3.3 Simulations and model validations

In this section, we demonstrate how the toolbox can simulate real-world flow reactors and validate our derived and implemented models using measurements. The experimental data used for validation was recorded using various PAT tools, including inline FTIR, HPLC, and UV-Vis spectroscopy. Data from these sources was processed using Partial Least Squares (PLS) models, which were trained on calibration samples covering relevant concentration ranges of reactants and products. Spectral pretreatment included baseline correction and derivative filtering, and the resulting models were used to convert measured spectra into accurate concentration profiles. Separate PLS models were applied for each experiment to reflect the specific system composition. The resulting concentration and condition data were imported into MATLAB using the import tool, synchronized to a common time base, and resampled to ensure consistent sample intervals across all signals. Finally, the cleaned and preprocessed data were saved as *.mat* files. Detailed information on the data preparation process can be found in the SI.

#### 3.3.1 Model validation of delay and dispersion effects

**Fig. 7:**
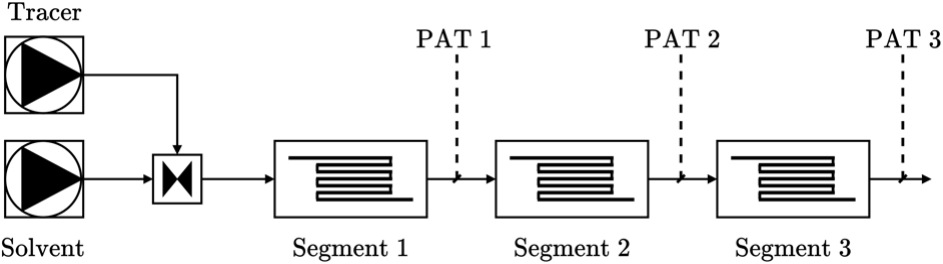
Illustration of the experimental setup to conduct the tracer experiments.

We conducted several tracer experiments, in which a tracer dissolved in a solvent flowed through a reactor setup consisting of three segments. The feed solutions were delivered using Knauer AZURA P 4.1S HPLC pumps (10 mL min^−1^ pump head, Hastelloy/ceramic, equipped with pressure sensors). The concentration of the tracer was measured after each segment using different process analytical tools (PAT), including FTIR, UHPLC, and UV-Vis. FTIR (Fourier-Transform Infrared Spectroscopy) is used to identify and quantify chemical compounds based on their absorption of infrared light. UHPLC (Ultra-High-Performance Liquid Chromatography) separates and quantifies components in a mixture, offering high resolution and fast analysis. UV-Vis (Ultraviolet-Visible Spectroscopy) measures the absorption of light in the ultraviolet and visible ranges, providing information about the concentration of analytes in solution. Each segment comprised of a tube, with the lengths and diameters of the tubes varied across the experiments. Additionally, the flow rate was modified throughout different experiments. The described setup is illustrated in Fig. 7.

Within our toolbox FlowMat, we can model the reactor setup. Here, we can choose one of the different modeling approaches or combinations of them to model the tubes within the three segments. As the tracer flowing through the pipes will only experience delay and dispersion effects, the approach using transfer functions is the most lightweight one. Besides rebuilding the reactor setup, we can import the measurements from the experiments, to use the real flow rates of the pumps and directly, enabling direct comparisons between the measured and simulated tracer profiles after each segment.

**Fig. 8:**
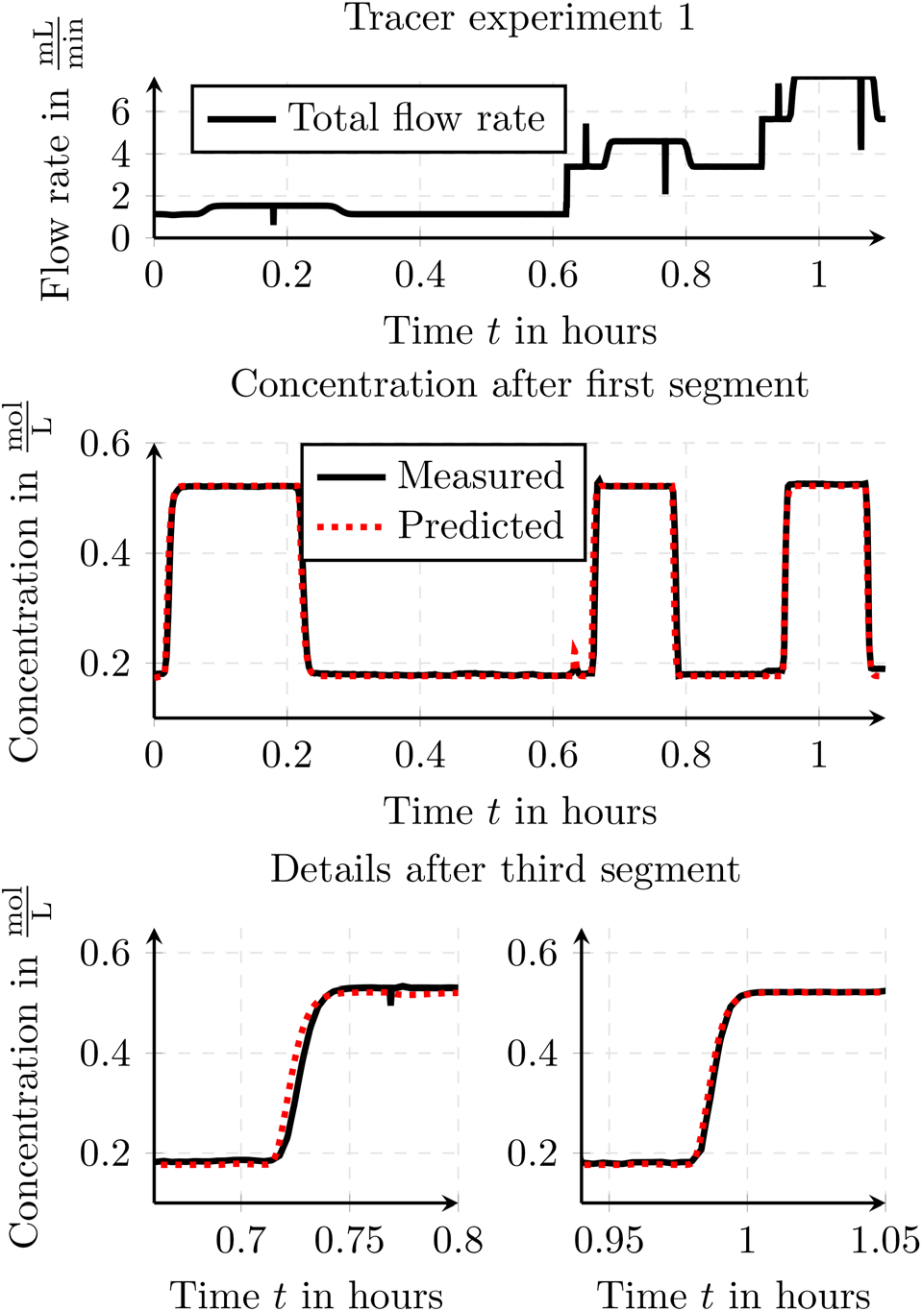
Comparison of the measured and simulated tracer values from the first tracer experiment.

Fig. 8 compares the measured and simulated tracer values from the first tracer experiment. In the first experiment, the tube lengths and diameters remained constant over time for each segment, while the flow rates were varied. To model the tubes, the modeling approach of transfer functions was applied. The time constants *T*_1_ and *T*_2_ were manually adjusted during the initial use of a tube to ensure alignment between the simulated values and the measured data. The time constants represent the dynamic response of the system and capture the dispersion behavior of the tracer within the tubing. While *T*_1_ and *T*_2_ were empirically adjusted in the present study to achieve good agreement with experimental data, they are physically motivated and could be estimated based on tube geometry and flow characteristics. The toolbox also allows for optimizing these parameters based on step response experiments, or they can be estimated using correlations from literature. Additionally, Fig. 6 in Section 3.2.1 illustrates the influence of *T*_1_ and *T*_2_ on a representative step response and can serve as a guide for parameter selection. The overall effort for identifying suitable parameters is low, typically requiring only one or two short tracer experiments per tube, making the approach practical for routine use. While an optimization based on experimental data may require more effort, it can yield more accurate results tailored to specific setups. Subsequently, these time constants were reused in other experiments involving the same tube to demonstrate their applicability and consistency across different experiments. Since the dispersion effects vary with different flow rates, several anchor points were used to define the time constants for some of those flow rates. Between these anchor points, the time parameters of the transfer function were linearly interpolated.

The results seen in the figure demonstrate that the overall concept of simulating delay and dispersion effects is valid, as the simulated and measured traces show good agreement. Additionally, the results clearly demonstrate the validity of the interpolation method between two anchor points. The slight peak observed in the concentration after the first segment of the model prediction around *t* = 0.6 hours can be attributed to minor inaccuracies during the transition between pump settings at the beginning of a new tracer step. These transient fluctuations cause a short-lived disturbance in the tracer profile. The peak is apparent in the simulation, as the raw pump data is used as input.

The identified parameters of the tubes used can be stored for experiments involving the same tube within a segment. This allows for reusing parameters of previously identified tubes, requiring only the characterization of new tubes.

#### 3.3.2 Model validation of a flow reactor including reactions

To further validate the implemented models of the toolbox, we simulate a flow reactor in which a reaction takes place. For the first validation, we make use of the Paal–Knorr reaction with one reaction. The Paal–Knorr reaction with one underlying reaction, involves three key species:

• the first species *C*_1_(*t*) is iso-propanol, acting as solvent,

• the second species *C*_2_(*t*) is ethanolamine (NH_2_–CH_2_–CH_2_OH) at a concentration of 1.5 mol L^−1^, and

• the third species *C*_3_(*t*) is 2,5-hexanedione (C_6_H_8_O_2_) at 1.5 mol L^−1^.

**Fig. 9:**
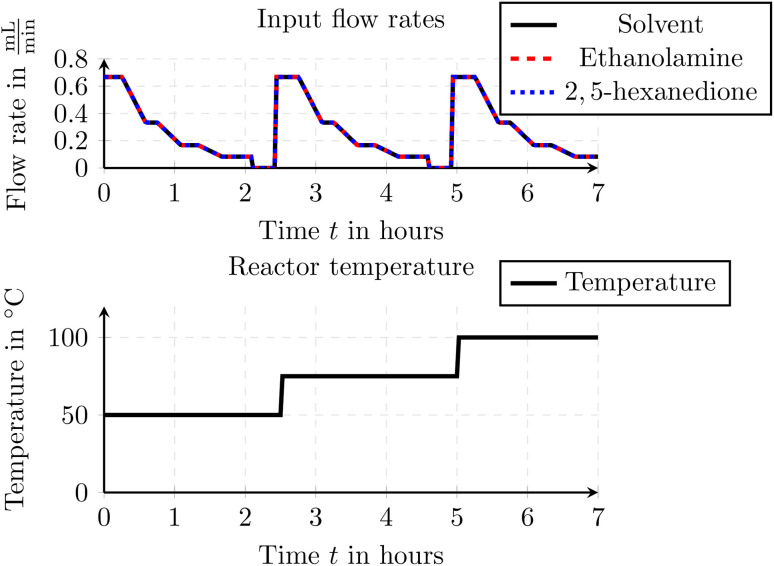
Input flow rates and reactor temperature for the Paal–Knorr reaction with one reaction.

The experimental setup consisted of pumping all three components into a 5 mL flow reactor under controlled flow rates and temperature conditions. Feed solutions were delivered using Knauer AZURA P 4.1S HPLC pumps (10 mL min^−1^ pump head, Hastelloy/ceramic, equipped with pressure sensors). To maintain consistent system pressure, a back pressure regulator (BPR, Upchurch, P-465) with a 34 bar (green, P-765) cartridge was installed directly downstream of each HPLC pump. The inlet streams were combined using a 7-port mixer (3 ports blocked with blanks), and the flow reactor was assembled with PFA tubing (0.8 mm i.d.) and thermostated using a Huber Ministat 240. The reaction mixture was continuously monitored using inline FTIR spectroscopy (Mettler Toledo React IR 15) with a DS Micro Flow Cell Diamond flow cell. Downstream of the FTIR, a membrane-based BPR (Zaiput BPR-10) set to 5 bar was integrated to regulate pressure within the reactor. Details on the selected input flow rates and reactor temperature are provided in Fig. 9.

The HPLC pumps and the thermostat were integrated into the experimental setup *via* RS232 connections to the HiTec Zang LabManager. The flow rate and temperature ramps were programmed using HiText (HiTec Zang), with setpoints configured in the LabVision software (HiTec Zang). Inline FTIR spectra were acquired using a Mettler Toledo ReactIR 15 equipped with a DS Micro Flow Cell Diamond. Data points were recorded every 15 seconds, with spectra captured between 4000 and 600 cm^−1^ at a resolution of 4 cm^−1^. The spectra were exported using iCIR7 software and automatically processed with a PLS model in Peaxact Process Link (S-PACT), ensuring efficient and accurate data analysis.

**Fig. 10:**
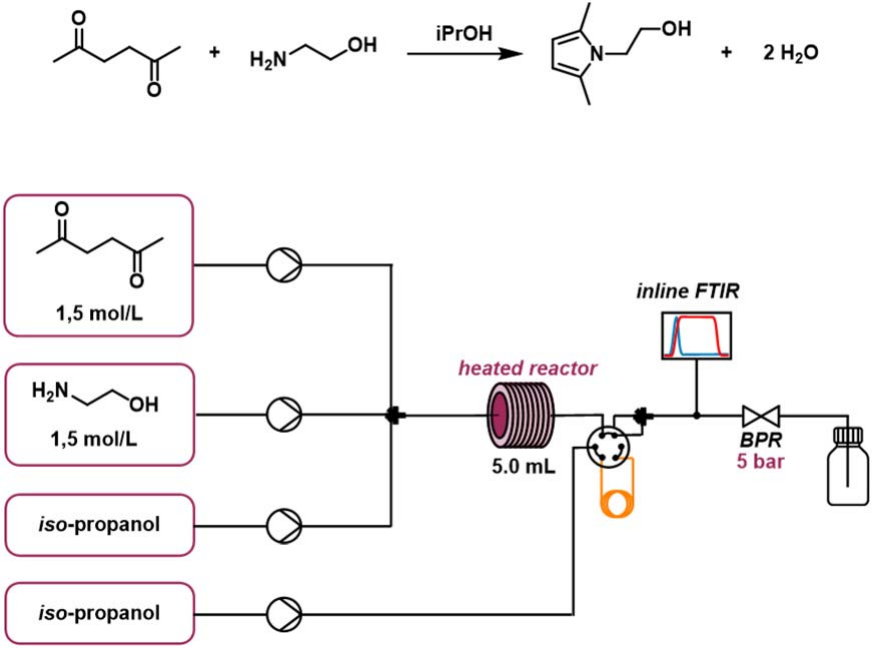
Reactor setup and stoichiometry of the Paal–Knorr reaction with one reaction.

Within the reactor, ethanolamine reacts with hexanedione to form the final product (1-(2-hydroxyethyl)-2,5-dimethylpyrrole) along with two molecules of water. The final product is referred to as species *C*_4_(*t*). The concentrations of the final product and residual materials are measured after the flow reactor. To allow the cleaning of the used PAT, an additional path was added in which the solvent is pumped through the end part of the reactor allowing to flush any residual material out of the sensor of the FTIR. One can find the reactor setup and the stoichiometry of the Paal–Knorr reaction with one reaction in Fig. 10.

In our Simulink model we can make use of the tanks-in-series model, the axial-dispersion model or data-driven approaches to include reactions within our reactor model. For the first validation including one reaction, we make use of the axial-dispersion model where we use the parameters found by the external software solution Dynochem.^40^ Dynochem is a process simulation and optimization software developed by *Scale-up Systems*, widely used in the pharmaceutical and chemical industries for modeling and optimizing batch and continuous manufacturing processes. It can also be used to identify parameters, supporting rapid scale-up, troubleshooting, and process development to enable efficient and reliable production workflows. After entering the parameters and importing the experimental data, we can simulate the flow reactor using the same flow rates as for the real-world experiment. After the simulation, we can directly compare the results with the measurements from the experiments.

**Fig. 11:**
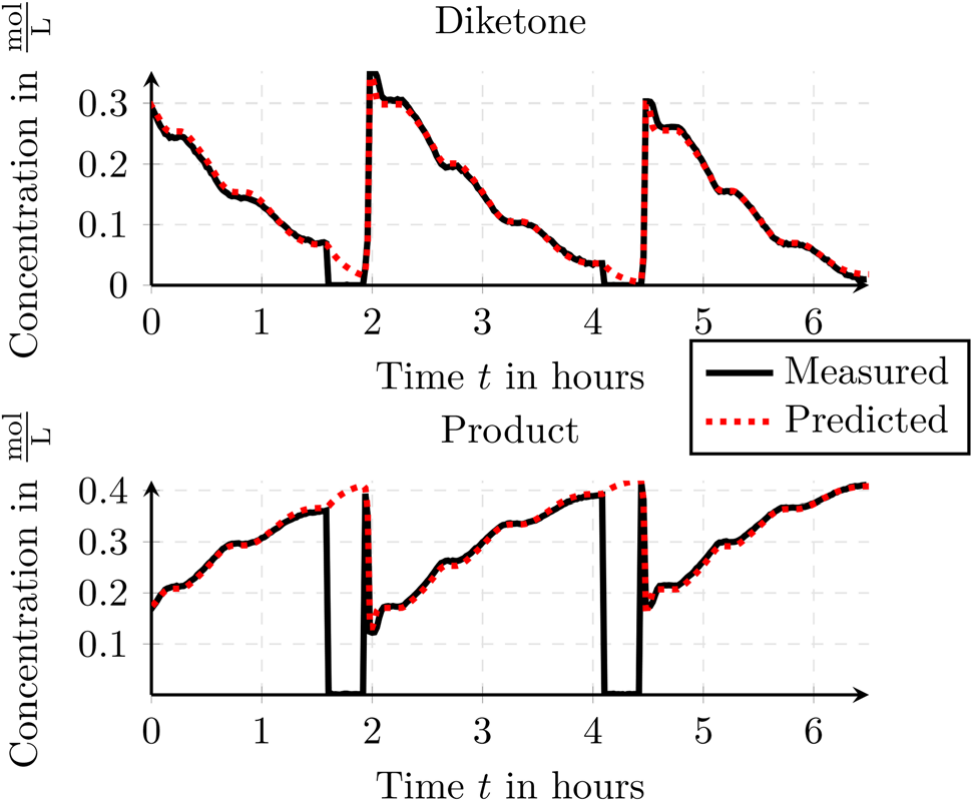
Comparison of the measured and simulated species concentrations for the Paal–Knorr reaction with one reaction using the axial-dispersion model. The concentration drops to 0 are attributed to the FTIR being rinsed with isopropanol.

**Fig. 12:**
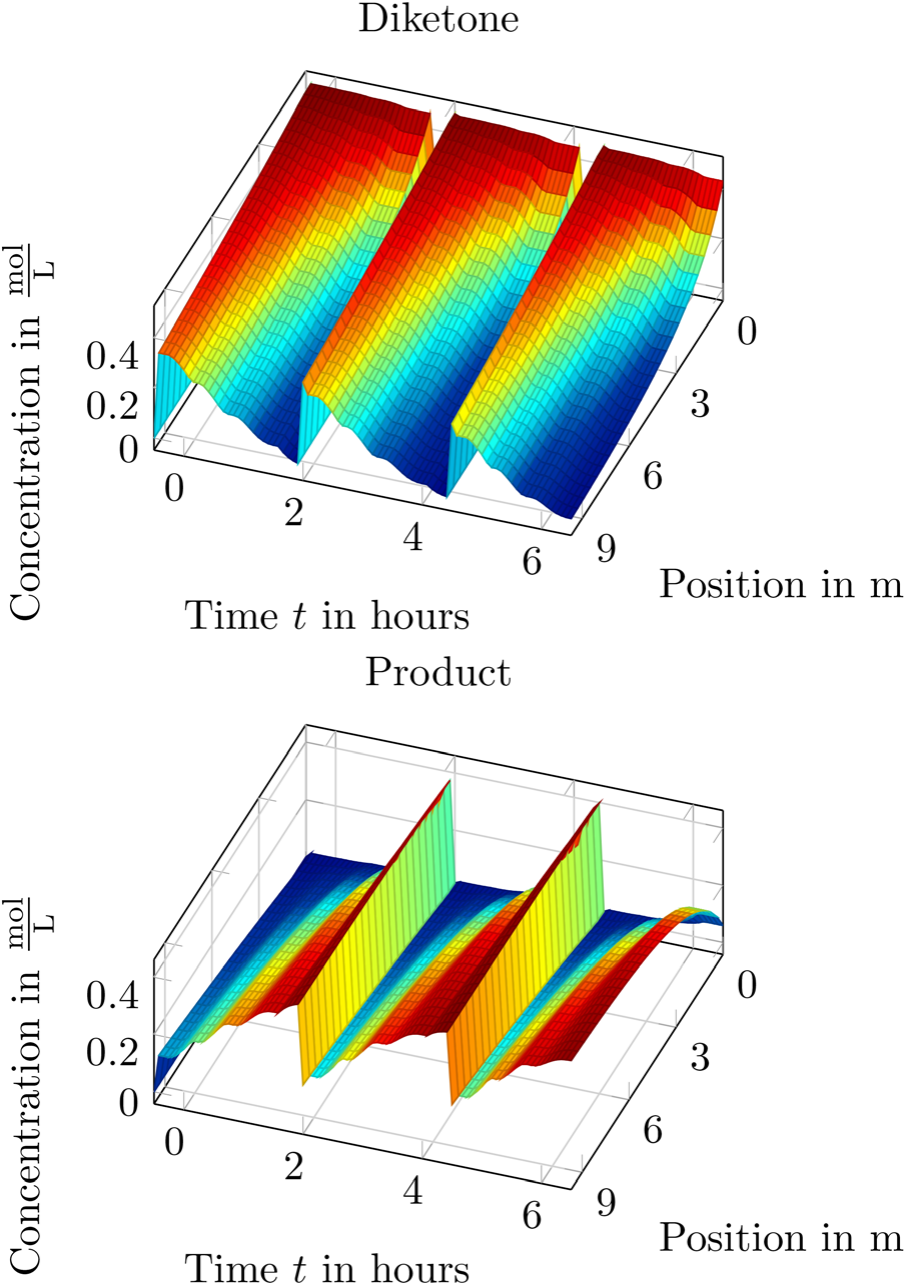
Spatial reactor insight of the Paal–Knorr reaction with one reaction using the axial-dispersion model.

In Fig. 11 the measured and simulated traces for the formed product and the remaining starting material (diketone – *C*_3_(*t*)) is depicted. As one can see, the simulated flow reactor depicts the real-world behavior well and thus validating the results of the toolbox when using the axial-dispersion model. As the axial-dispersion model is comparable to the tanks-in-series model as we can set the axial-dispersion coefficient *D* = 0, we can also state, that the simulation can be used to validate the tanks-in-series model. Additionally to the outflowing concentrations, the toolbox can also deliver the spatial information within the reactor which is depicted in Fig. 12. Thus it is possible to immediately see how the species are consumed and formed throughout time and space, which might be a useful insight when designing the reactions and the reactor setup.

Besides the PBM, one can also use DDM to model those reactor systems such as a neural network. Thus we used the toolbox to train a shallow fully connected neural network using the experimental data. As mentioned in Section 3.2.4, one can split the available data into a training set and a validation set to evaluate the generalization performance of the neural network. In this example, 70% of the available data was used for training, while the remaining 30% served as the validation set. This can be achieved by providing only the training portion of the data to the FlowMat training method of the neural network. To assess model performance, the mean squared error (MSE) was calculated for both subsets by summing the MSE values across all predicted concentration traces. This resulted in an MSE of 17 × 10^−3^ for the training set and 35 × 10^−3^ for the validation set. These values indicate a good fit to the training data while still preserving reasonable generalization to unseen data, as the validation error remains comparable to the training error. The neural network can afterwards be used within the simulation or directly within a MATLAB/Simulink script.

**Fig. 13:**
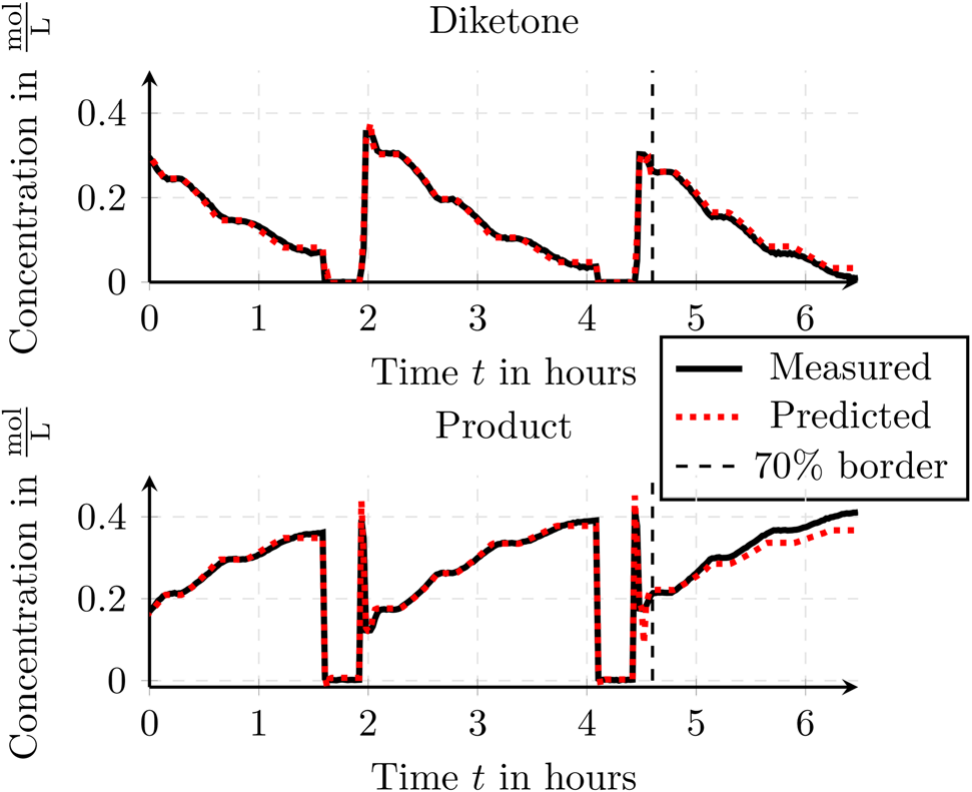
Comparison of the measured and simulated species concentrations for the Paal–Knorr reaction with one reaction using a shallow neural network. Data to the left of the 70% border was used to train the neural network.

The results of the simulation using the trained neural network are depicted in Fig. 13. In the figure, one can see the 70% split used for training (left section) and validation (right section) of the data. The figure visually confirms that the trained neural network is able to predict the outflowing concentrations not only for the training data but also for the unseen validation data, demonstrating its generalization capability. This validates the method and shows that the method can be applied to simulate a real-world process.

### 3.4 Parameter identification using FlowMat

Most of the time, the underlying reactor and reaction parameters are not known. Thus, we want to highlight the possibility, to estimate parameters using our toolbox. With FlowMat, it is possible to optimize the parameters such that the simulated values match the measured ones. It is possible to define constraints on those parameters such that they stay in physically feasible bounds. The optimization of parameters can be conducted in different ways. One can use PBM, such as the tanks-in-series model, or the axial-dispersion model, or DDM, such as physics-informed neural networks. For the DDM method, a neural network is extended to a physics-informed neural network whereby the parameters are an additional output when training the physics-informed neural network with experimental data. Additionally, we want to point out, that it is possible to use transient data for the estimation of the reaction parameters. The recording of transient data is more cost effective and faster compared to recording of steady-state data as the information between two steady-state points is already used.

For the identification of reaction parameters we investigated the Paal–Knorr reaction with two reactions. The Paal–Knorr reaction, with two consecutive reaction steps, involves three key species:

• the first species *C*_1_(*t*) is the solvent, a mixture of toluene and methanol in a 2 : 1 ratio,

• the second species *C*_2_(*t*) is ethylenediamine (NH_2_–CH_2_–CH_2_–NH_2_) at a concentration of 0.75 mol L^−1^, and

• the third species *C*_3_(*t*) is 2,5-hexanedione (C_6_H_8_O_2_) at 1.5 mol L^−1^.

**Fig. 14:**
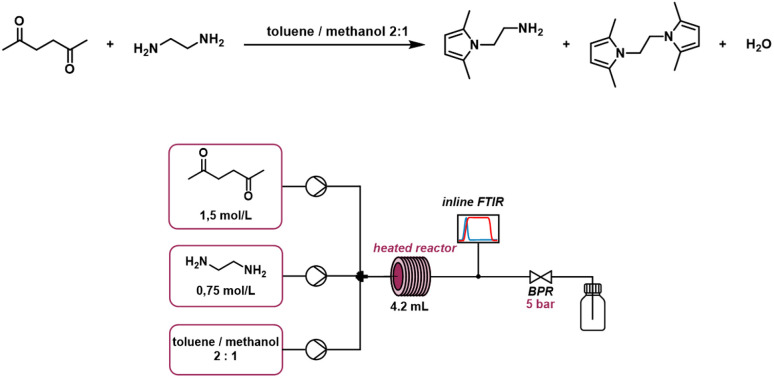
Reactor setup and stoichiometry of the Paal–Knorr reaction with two reactions.

All three components are pumped into a 4.2 mL flow reactor at controlled flow rates. The same equipment as for the Paal–Knorr reaction with one reaction was used, including Knauer AZURA P 4.1S HPLC pumps, back pressure regulators (BPR), and inline FTIR spectroscopy (Mettler Toledo ReactIR 15). Within the reactor, ethylenediamine reacts with hexanedione to form an intermediate product. This intermediate product reacts further with hexanedione to produce the final product (2,5-dimethyl-1*H*-pyrrol-1-yl) ethane. The intermediate product and the final product are referred to as species *C*_4_(*t*) and species *C*_5_(*t*) respectively. The concentrations of the final product and residual materials are measured after the flow reactor. The reactor setup and the stoichiometry of the Paal–Knorr reaction with two reactions is depicted in Fig. 14.

It is assumed that both reaction steps follow the Arrhenius equation for which the reaction rates are given by25
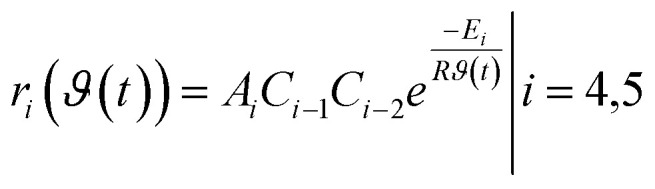
whereby the reaction parameters *A*_*i*_, *E*_*i*_ remain unknown. Thus, the goal is to use our toolbox to estimate those reaction parameters using different methods and make use of experimental data. The estimated reaction parameters can afterwards be used for simulation, validation and optimization of the reactor setup and the operation points or used for further analysis.

#### 3.4.1 Parameter identification using a PBM and transient data

The first option to identify underlying reactor parameters is to use PBM. Thereby, the tanks-in-series model or the axial-dispersion model and the inbuilt MATLAB/Simulink optimization functions are used. As mentioned, it is possible to define constraints for the parameters such that they stay in feasible regions, and one can speed up the optimization by providing feasible initial values. In general, all available MATLAB optimization algorithms can be utilized along with third-party optimizers, such as YALMIP.^41^ In this study, we employed the MATLAB *fmincon* function, which supports various algorithms, including ‘*interior-point*’, ‘*sqp*’, ‘*active-set*’, and ‘*trust-region-reflective*’. Each algorithm offers distinct advantages depending on the problem structure, whereby we selected ‘*interior-point*’ which is designed for solving large-scale constrained optimization problems and works efficiently with both linear and nonlinear constraints.

**Fig. 15:**
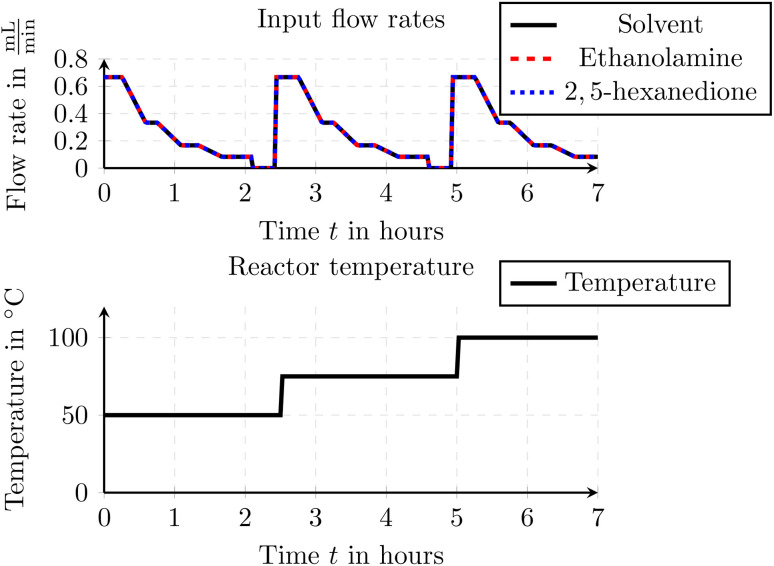
Transient input flow rates and reactor temperature for the Paal–Knorr reaction with two reactions.

For the optimization we used an experiment with transient data. The use of transient experimental data eliminates the need to wait for steady-state conditions, thereby reducing overall time, material consumption, and labor. This makes the parameter identification and optimization process significantly more efficient. In the experiment the flow rates and temperature profiles were varied throughout the entire experiment and can be found in Fig. 15. Due to the transient input data, also the outflowing concentrations show a continuous change and never reach steady state. This typically makes it harder to estimate proper reaction parameters. Traditional identification methods do not consider underlying dispersion and delay effects and thus the parameters are often not estimated correctly. As we consider those delay and dispersion effects within our models and within our optimization of the parameters we can also make use of transient data and we are not limited to steady state data.

For the Paal–Knorr reaction with two reactions, we model the reactor setup using the axial-dispersion model. In the axial-dispersion model we defined the reaction parameters to be variables which can be optimized. Using the imported experimental data, we can use the used flow rates as input and optimize the parameters in such a way, that the simulated concentration outputs align with the measured outputs from the imported experiment. As the experimental data might show phases which are not representative, the toolbox allows to specify time intervals which should be used for the optimization.

**Fig. 16:**
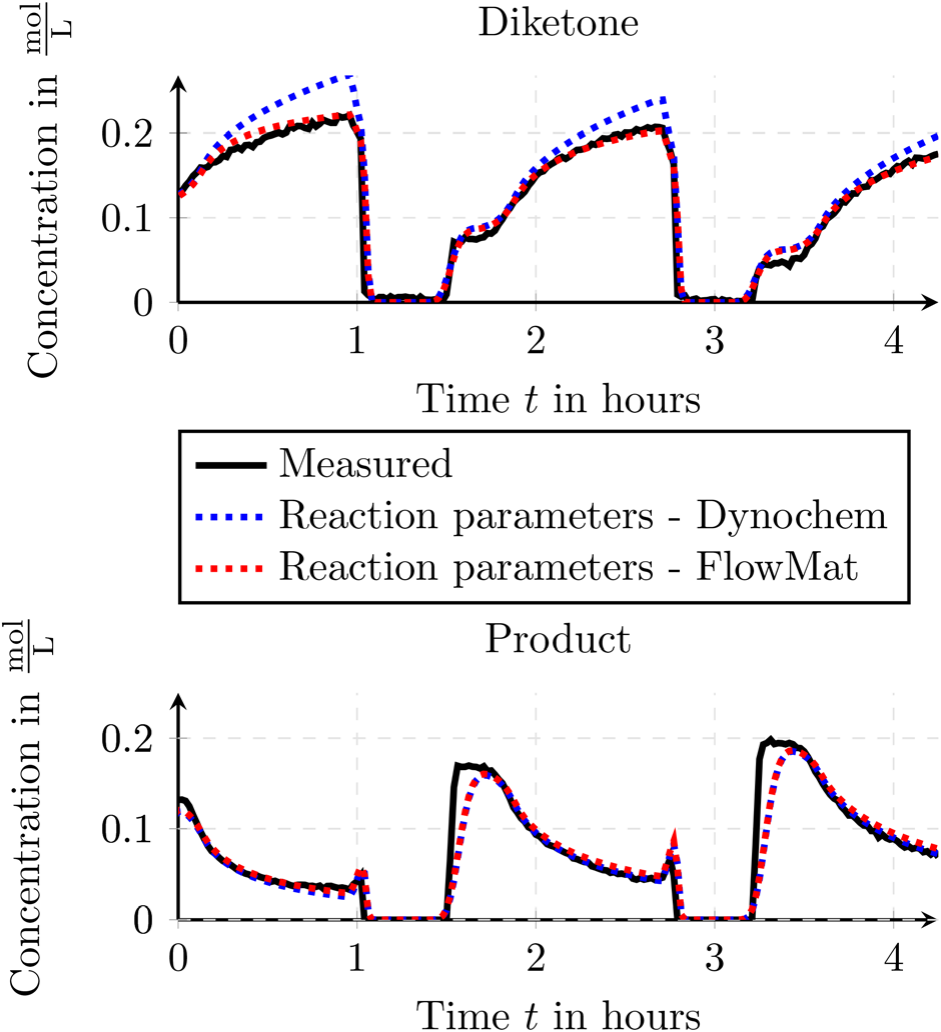
Comparison of the measured and simulated species concentrations for initial parameters and optimized parameters for the Paal–Knorr reaction with two reactions.

In Fig. 16 we depicted the results after the optimization of the initial reaction parameters found by the external software solution Dynochem. In the figure, one can see the prediction using the reaction parameters found by the external software solution Dynochem and the predictions after the optimization from FlowMat. The simulations for the reaction parameters from Dynochem already show useable predictions yet after the optimization, the predictions agree much better with the measured data.

**Fig. 17:**
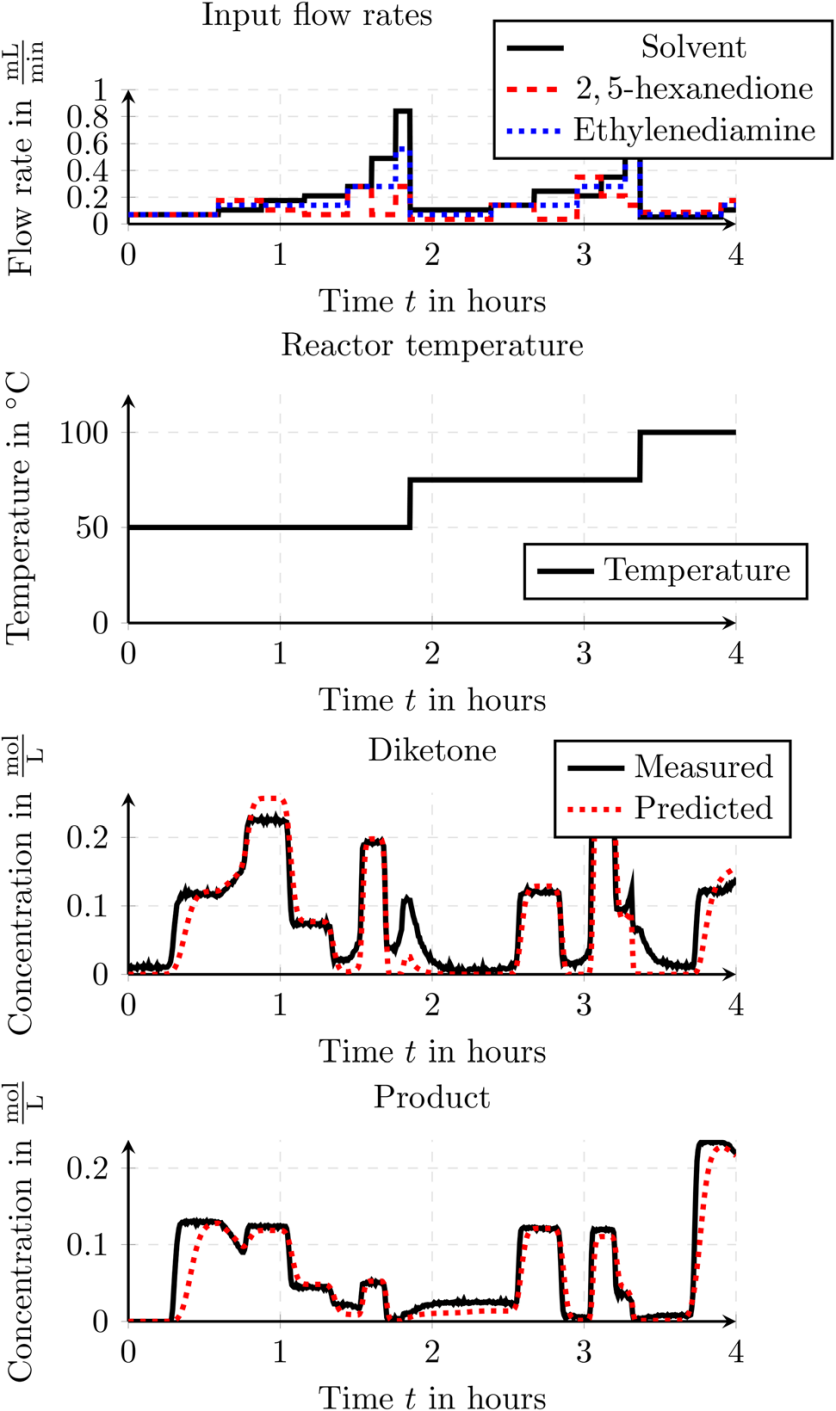
Comparison of the measured and simulated concentrations for an experiment with steady state traces for the Paal–Knorr reaction with two reactions. The parameters were estimated using an experiment with transient data which shows huge differences in the actuation patterns.

As we used transient data to find the reaction parameters, we want to use the estimated parameters to cross test them with an unseen experiment. In Fig. 17 one can see the inputs and the prediction results when we test the found parameters on an additional experiment. As one can see, the second experiment shows huge differences in the actuation patterns and shows more steady state intervals and less transient phases compared to the experiment which was used to identify the reaction parameters. As seen in the figure, the predicted output agrees with the measured data indicating, that the estimated parameters can also be used to predict the output concentrations for the same flow reactor setup when using different input data.

#### 3.4.2 Parameter identification using physics-informed neural networks

Using FlowMat, we can also make use of physics-informed neural networks to estimate physical parameters. To do so, the toolbox allows to define a custom training function when training a neural network. In the custom training function we can define additional loss factors and incorporate physical models such as the axial-dispersion model. Necessary and unknown parameters within the axial-dispersion model can be estimated by the physics-informed neural network. The output estimations of the physics-informed neural network are extended as described in Section 3.2.5 to include the spatial predictions of each concentration and additional estimates for the unknown parameters. In the custom training function, one can make use of those estimates and calculate the losses given by the measured data and the axial-dispersion model.

**Fig. 18:**
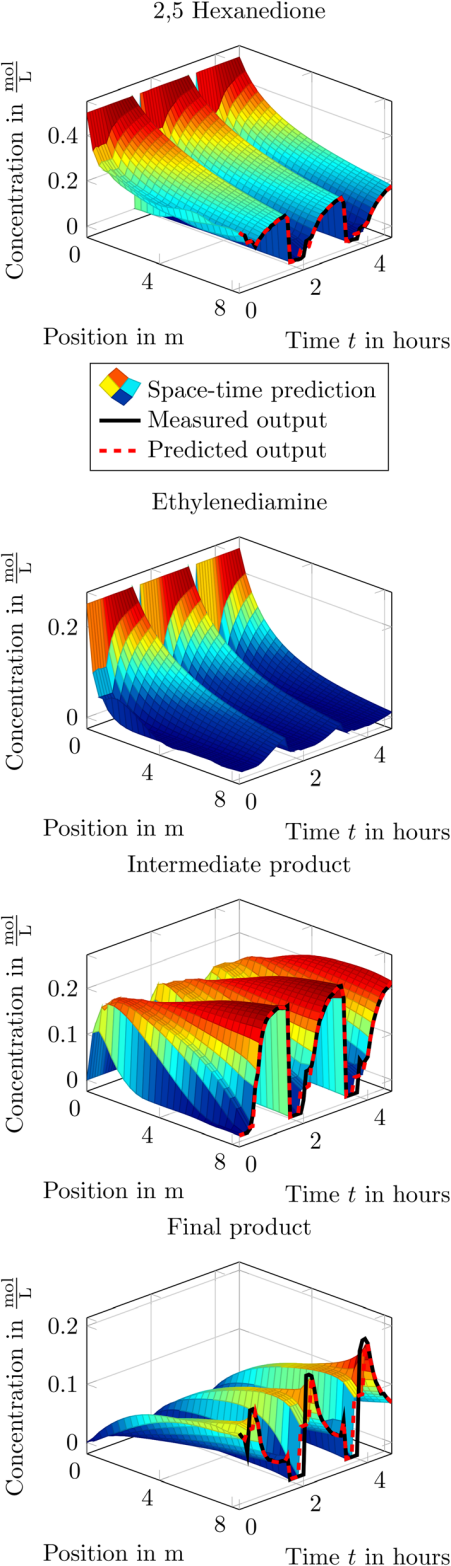
Time-space result of the physics-informed neural network after training for the Paal–Knorr reaction with two reactions.

When applying the training of a physics-informed neural network to estimate the reaction parameters to the introduced Paal–Knorr reaction with two reactions we find comparable parameters like in Section 3.4.1. Additionally, the resulting physics-informed neural network can be used to gain insight in the time-space behavior within the flow reactor. In Fig. 18 the time-space results of the physics-informed neural network after training are depicted. Additionally, the measured concentrations are depicted in black at the reactors output along with the predicted output concentration of the physics-informed neural network in red. As one can see, both traces agree within all concentrations quite well. Moreover, the time-space results are comparable to those of the axial-dispersion/tanks-in-series model and verify that the underlying axial-dispersion model is incorporated.

#### 3.4.3 Parameter validation

To evaluate and compare the performance of the three modeling techniques—Dynochem as an external software solution, the axial-dispersion model, and the physics-informed neural network within FlowMat—we aim to compare each approach using the experiment which was used for the parameter identification. The comparison will be based on an estimation error for all three techniques. Specifically, we define the estimation error *ζ*_p_ as:26
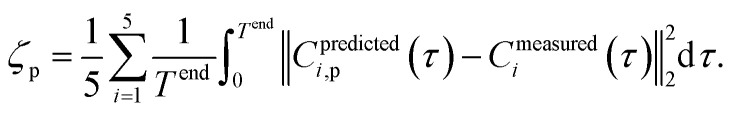


In the equation, *p* denotes the modeling technique, and the found estimation errors *ζ*_p_ are summarized in Table 1. The estimation error *ζ*_p_ is calculated as the sum of the squared differences between the measured values *C*^measured^_*i*_(*τ*) and the predicted values *C*^predicted^_*i*,p_(*τ*) across all concentrations *i*. This sum is then normalized with respect to the number of concentrations *P* = 5 and the total experimental duration *T*^end^.

**Table 1:** Estimation errors *ζ*_p_ for the three modeling techniques

Modeling technique *p*	Estimation error *ζ*_p_
Dynochem	4.17 × 10^−4^
Axial-dispersion model (FlowMat)	2.45 × 10^−4^
Physics-informed neural network (FlowMat)	0.88 × 10^−4^

Both FlowMat techniques—the axial-dispersion model and the physics-informed neural network method—demonstrate smaller estimation errors *ζ*_p_ compared to the external software solution Dynochem. Notably, the physics-informed neural network method achieves the lowest estimation error *ζ*_p_, highlighting its status as the most advanced modeling approach available. However, this technique is accompanied by increased complexity. Despite this higher complexity of the physics-informed neural networks but also the axial-dispersion model, FlowMat offers a user-friendly interface that makes these advanced methods, remarkably easy to use. The identified results highlight that the more complex modeling techniques within FlowMat outperform the external solution, thereby proving the usability and effectiveness of the toolbox's modeling techniques.

To further validate the estimated reaction parameters, we want to cross test the parameters using one modeling technique. Given the reaction parameters **Γ**_p_ = {*A*_4_, *A*_5_, *E*_4_, *E*_5_} from the software solution Dynochem (parameter set **Γ**_Dyno_), the identified reaction parameters using the axial-dispersion model (parameter set **Γ**_AD_) and the reaction parameters identified using a physics-informed neural network (parameter set **Γ**_PINN_), we want to validate and compare each parameter set **Γ**_p_ using the axial-dispersion model. Our goal is to demonstrate that all three techniques for identifying reaction parameters yield comparable results, which can also be utilized in other modeling approaches.

We tested the identified reaction parameters **Γ**_p_ for the introduced reactor setup on various experiments. Besides the experiment with transient data (Experiment *l* = 1 – which was used for identification of the parameters and thus serves as a reference), we also used the experiments showing more steady-state phases (Experiment *l* = 2 – which was also used in Section 3.4.1 to verify the identified reaction parameters from transient data on steady-state data) for validation. The flow reactor setup using the axial-dispersion model was simulated for the various reaction parameters for all available experiments. Afterwards we determined the overall estimation error27
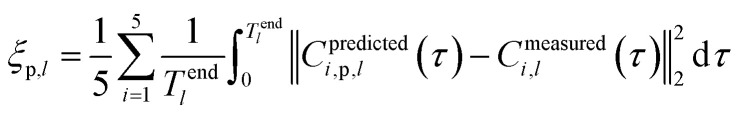
for each combination. In eqn (27), *C*^predicted^_*i*,p,*l*_(*τ*) denotes the predicted concentration of species *i* for experiment *l* when using the reaction parameters **Γ**_p_. The term *C*^measured^_*i*,*l*_(*τ*) denotes the measured values from experiment *l* for species *i*. The term *T*^end^_*l*_ represents the according end time of experiment *l*.

**Fig. 19:**
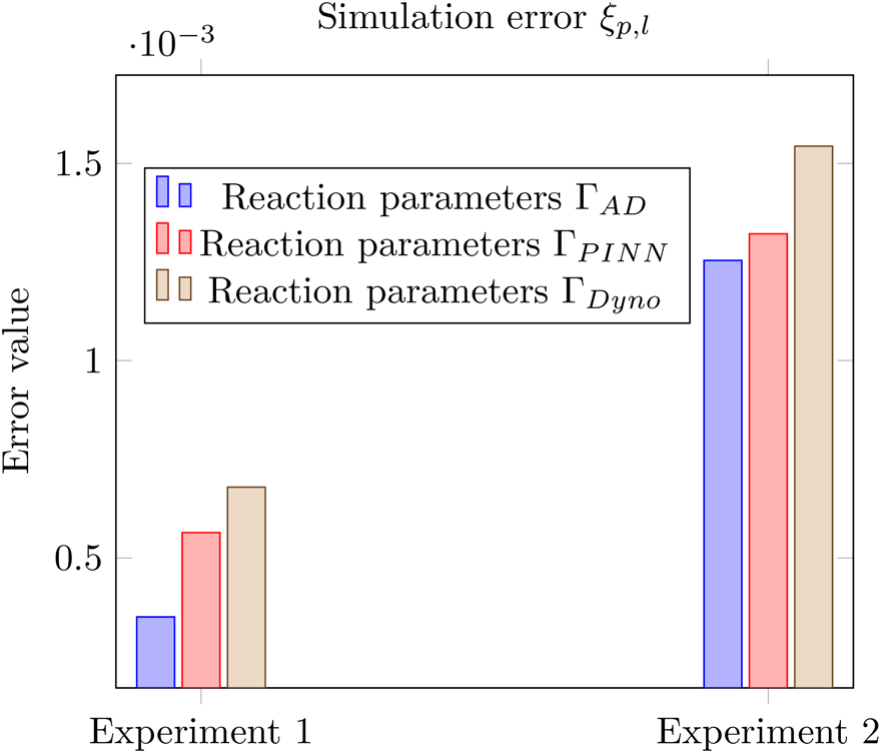
Simulation error *ξ*_p,*l*_ for all experiments *l* and reaction parameters **Γ**_p_. The first experiment (*l* = 1) was used for parameter identification, with all simulations conducted using the method of the axial-dispersion model. Consequently, the simulation based on the first experiment and the axial-dispersion model shows the smallest error and serves as a reference.

The results of the overall estimation error are depicted in Fig. 19. In the figure, one can see, that all sets of reaction parameters **Γ**_p_ result in similar estimation errors *ξ*_p,*l*_ throughout all experiments *l*. It is obvious, that the first experiment *l* = 1 shows the smallest overall simulation errors *ξ*_p,*l*_ for all reaction parameters as the experiment was used for the identification. Thus the values for the first experiment can be used as a reference. The other experiments show larger values yet are in a comparable range. Despite the small increase in the overall simulation error when cross testing the found parameters, it is essential to see, that the simulation error values *ξ*_p,*l*_ within an experiment *l* show a similar performance. We also want to highlight, that for experiment *l* = 2 the reaction parameters found by our toolbox FlowMat outperform the reaction parameters found by the external software solution Dynochem. Moreover, one can conclude, that all parameters seem to be a valid choice when facing the small gap of the simulate error within one experiment *l* and the comparable performance for unseen experiments.

### 3.5 Optimization

Having identified the appropriate parameters and validated the simulation results of the reactor system, one can consider optimizing both: the operational points and the reactor setup itself. To do so, we use our toolbox in combination with the built-in MATLAB/Simulink optimization tool. Again, all available MATLAB optimization algorithms can be utilized along with third-party optimizers. For the optimization of the reactor, we employed the MATLAB *fmincon* function, whereby we selected the ‘*interior-point*’ algorithm. Using the built-in optimization, FlowMat enables the optimization of operating points to determine ideal temperatures and input flow rates based on a customizable objective. Additionally, reactor parameters such as reactor length *L* and reactor diameter *d* can be optimized while respecting specified constraints. The optimization allows for the definition of multiple objectives, which can either be combined into a single target function or used to search for the pareto front. The pareto front represents a set of solutions where no single objective can be improved without compromising another, offering valuable trade-off information for decision making.

We use our toolbox to optimize the operating conditions and the reactor setup for the introduced Paal–Knorr reaction with one side product and one desired product. Within FlowMat we modeled the given reactor setup and determined all necessary reaction parameters as described in Section 3.4. Given a validated reactor model of the real-world flow reactor setup, we can use it for the optimization. We introduce necessary optimization variables such as the flow rates *q*_*i*_ with *i* = 1, 2, 3 for all three input species (solvent, ethylenediamine and 2,5-hexanedione), the temperature *ϑ* within the reactor, and the total reactor volume *V*. We can collect the introduced optimization variables in a vector

28 **x** = [*q*_1_*q*_2_*q*_3_*ϑ V*]^*T*^.

The individual optimization variables can be used within the reactor model. The model can then be simulated for a defined time *T* using the given parameters in **x**. We denote the simulation of the reactor model with 
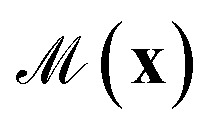
 which returns [*C*_1_(*t*) … *C*_5_(*t*)] where *C*_*i*_(*t*) represent the predicted output concentration of species *i*.

Regarding the objectives, one can, *e.g.*, think of

• maximizing the concentration of the final product *C*_5_(*t*) while minimizing the concentration of the intermediate product *C*_4_(*t*),

• maximizing the total flow rate 
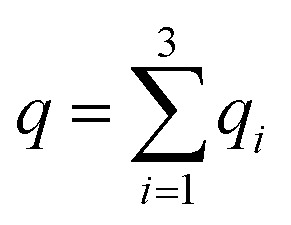
 to maximize the throughput,

• minimizing the temperature *ϑ* to reduce operational costs,

• minimizing the reactor volume *V* to reduce the nominal residence time and material costs.

As each of the optimization variables can only be within physically feasible bounds, we define the following constraints:

• the flow rates of the species must be between 
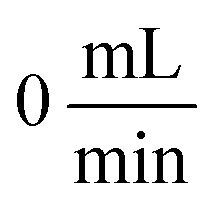
 and 
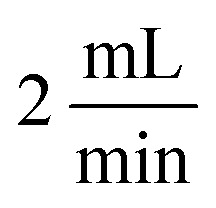
 whereby the flow rate of the solvent/species 1 must be greater than 
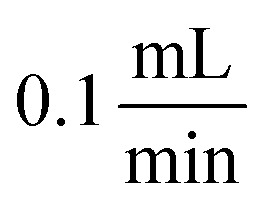
,

• the temperature *ϑ* must be between 20 °C and 200 °C,

• the total reactor volume *V* must be between 1 mL and 5 mL,

• the total flow rate *q* must be between 
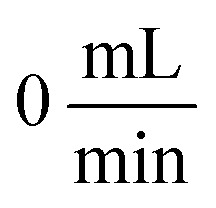
 and 
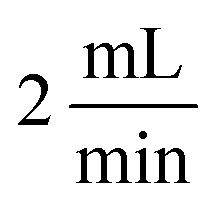
.

The optimization problem can afterwards be brought in the form of29
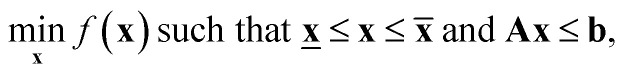
where *f*(**x**) is the objective or a combination of objectives like30
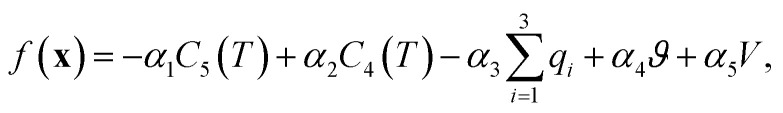
and31
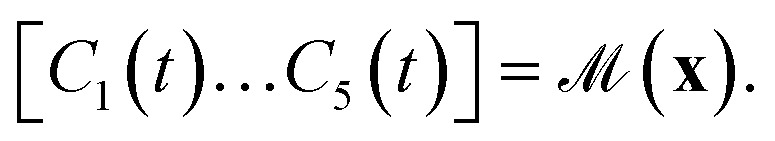


The factors *α*_*l*_ > 0 for *l* ∈ {1, …, 5} weight each individual term in the objective *f*(**x**) relative to the others. The term **x̲**, and **x̄** represent the lower and upper bounds of **x** respectively, and thus read as32
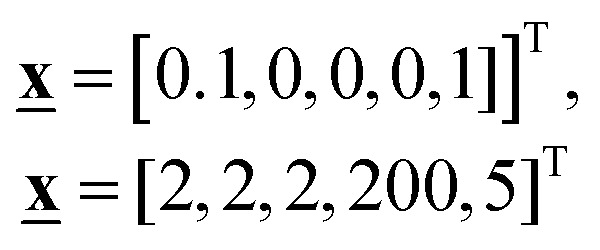
for our example. For the inequality constraints we can define

33 **A** = [1,1,1,0,0], **b** = 2

to realize, that the total flow rate *q* has to be within the upper limit of 
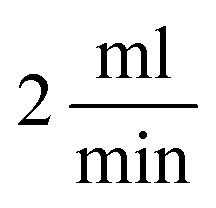
.

The found quantities of the optimization problem can easily be entered in our toolbox and allow an easy implementation of the optimization problem. For the weighting factor *α*_*l*_ one can choose a proper combination or use the possibility, to keep each objective separate and determine the pareto front using our toolbox.

**Fig. 20:**
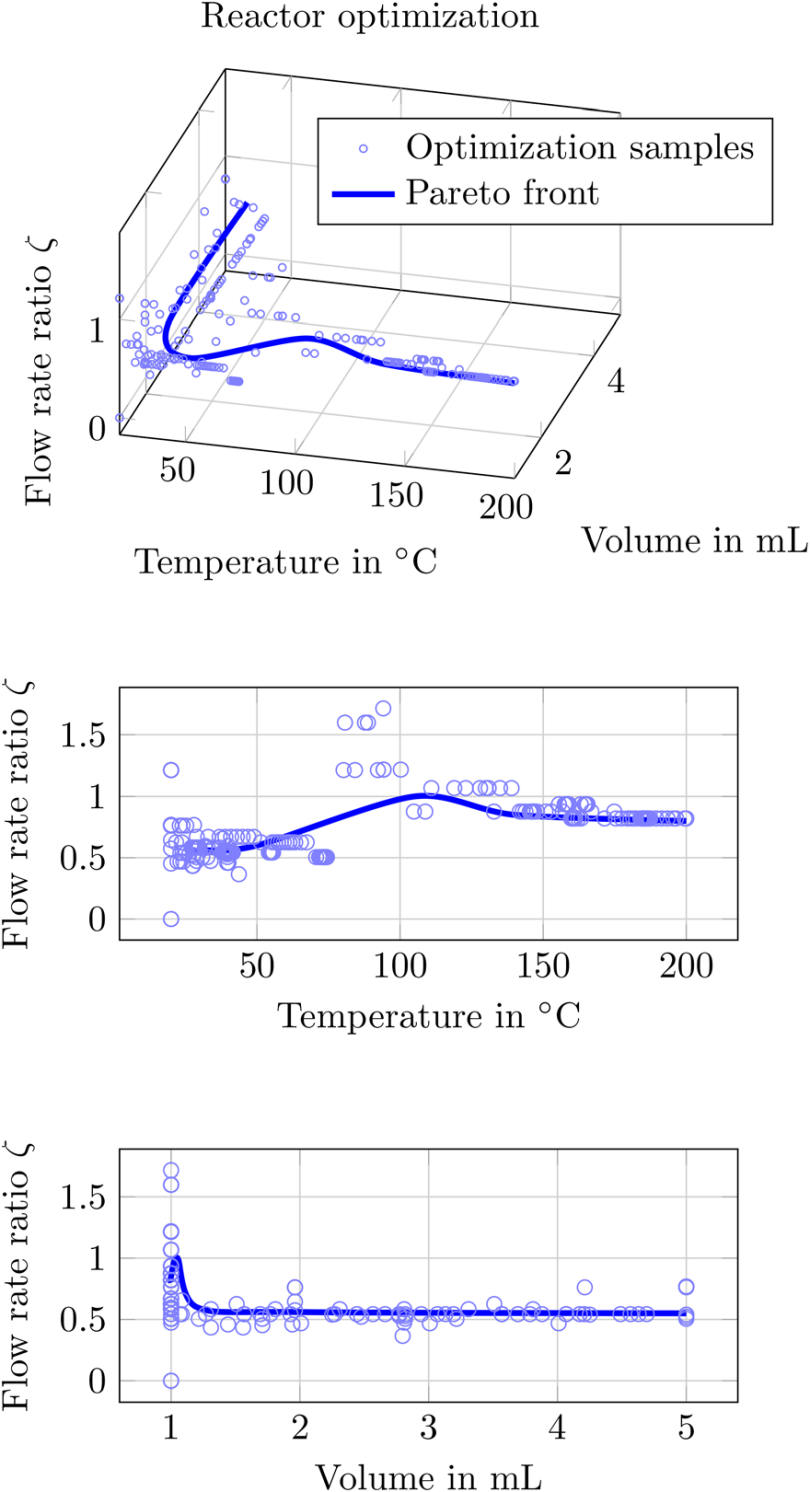
Illustration of the Pareto front for the optimization of a flow reactor setup. The three plots show the same data: the top is a 3D plot, while the two plots below represent 2D projections onto selected variable pairs for improved clarity.

In this paper we want to present the latter option whereby the resulting pareto front is found in Fig. 20. As we face several optimization variables the illustration is not trivial. Thus we depicted the ratio of the inflowing species 2 and 3 
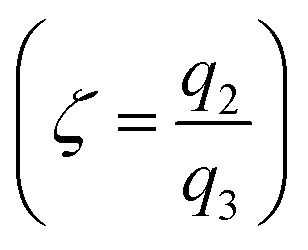
 which are consumed in the first reaction, the temperature *ϑ* and the reactor volume *V*. The total flow rate was always found to be at a maximum and thus giving no more insight when it was plotted.

In the depicted figure one can see, the pareto front for which no single objective can be improved without compromising another. When allowing higher temperatures *ϑ*, the total reactor volume *V* is reduced to a minimum while a very low temperature *ϑ* require a larger reactor volume *V*. This might be explained by the reaction rate being highly dependent on the temperature *ϑ* causing the desired product to be formed faster within a smaller volume for high temperatures and *vice versa*. Moreover, one can see how the optimal trace of the inlet ratio *ζ* changes throughout the temperature-volume space.

## 4 Conclusions

We developed FlowMat which is an accessible,^42^ open-source, yet powerful MATLAB/Simulink toolbox for modeling flow reactors. The toolbox is designed with a modular architecture, featuring an intuitive drag-and-drop interface that facilitates the reconstruction of real-world flow reactor systems. It supports various modeling approaches, including physics-based models (PBM), data-driven models (DDM) such as fully connected neural networks, and hybrid approaches like physics-informed neural networks (PINNs). These modeling methods can be used in parallel or individually, depending on the specific requirements of the application. While the implementation details of the modeling approaches are abstracted for ease of use, users have the flexibility to specify detailed parameters when needed.

After introducing the conceptual background, we demonstrated the toolbox's implementation and its ability to simulate real flow reactors, including chemical reactions. We further illustrated how the toolbox can be used to identify underlying parameters of the reactor and reactions by leveraging transient experimental data, enabling the determination of accurate reaction parameters that generalize to unseen data. As the toolbox allows the use of transient experimental data, there is no need to wait for the system to reach steady state, which generally reduces time consumption, material usage, and labor costs. This capability can thus contribute to lowering the overall cost and time associated with experimental parameter determination. Additionally, we employed advanced techniques, such as PINNs, to identify parameters, showing that the results were consistent and performed comparably.

Finally, we showcased the toolbox's potential for optimizing an entire reactor setup. Specifically, we determined the pareto front for a defined scenario, demonstrating its ability to balance multiple objectives and provide valuable insights for decision-making. FlowMat, represents a significant advancement in flow reactor modeling and optimization, offering robust tools for researchers and engineers in the field.

## Author contributions

Sebastian Knoll: conceptualization, formal analysis, methodology, validation, investigation, writing original draft, and visualization. Klara Silber: validation, investigation, and conducting experiments. Jason D. Williams: validation, investigation, supervision, and conducting experiments. Peter Sagmeister: investigation, supervision, and conducting experiments. Christopher A. Hone: validation, investigation, supervision, and conducting experiments. C. Oliver Kappe: resources, and supervision. Martin Steinberger: conceptualization, methodology, supervision. Martin Horn: resources, and supervision.

## Conflicts of interest

There are no conflicts to declare.

## Supplementary Material

RA-015-D5RA06173C-s001

## Data Availability

FlowMat is an open-source software and is fully available on GitHub (https://github.com/SKenb/FlowMat). Supplementary information: Experimental, hardware and software details and spectra. See DOI: https://doi.org/10.1039/d5ra06173c.
