# Supplementary material for: FlowMat: a toolbox for modeling flow reactors using physics-based and machine learning approaches for modular simulation, parameter identification, and reactor optimization
Source: RSC Adv. 2025 Sep 12;15(40):33278–96. doi: 10.1039/d5ra06173c (PMC12428333; doi:10.1039/d5ra06173c)
Supplement: RA-015-D5RA06173C-s001 [file RA-015-D5RA06173C-s001.pdf]

Supplementary Information (SI) for RSC Advances.
This journal is © The Royal Society of Chemistry 2025

FlowMat: A Toolbox for Modeling Flow Reactors using Physics-based and Machine Learning Approaches for a Modular Simulation, Parameter Identification, and Reactor Optimization

Sebastian Knoll,[a] Klara Silber,[bc] Jason D. Williams,[bc] Peter Sagmeister,[bc] Christopher A. Hone,[bc] C. Oliver Kappe,[bc] Martin Steinberger[a] and Martin Horn[a]

[a] Institute of Automation and Control, Graz University of Technology, Inffeldgasse 21b, 8010 Graz, Austria.

[b] Center for Continuous Synthesis and Processing (CCFLOW), Research Center Pharmaceutical Engineering GmbH (RCPE), Inffeldgasse 13, 8010 Graz, Austria.

[c] Institute of Chemistry, University of Graz, NAWI Graz, Heinrichstrasse 28, 8010 Graz, Austria

E-mail: martin.horn@tugraz.at; christopher.hone@rcpe.at

Inhalt

Toolbox – Details and Tutorials 3
- Introduction 3
- Availability 3
- Quantities and units 3
- FlowMat State 4
- Experiments 5
- Parts 6
- Examples / Tutorial 9
  - MATLAB/Simulink 9
  - FlowMat 9
  - Setup with an Axial dispersion model / Tank in Series model: 11
  - Reduced Paal-Knorr Reaction 15
  - Paal-Knorr Reaction 18
  - Paal-Knorr Reaction – Parameter optimization 19
  - Neural Networks 22
  - Physics-Informed Neural Networks 24

Reactor setup 25
- Reactor 25
- Automation 25
- Process Analytical Technology (PAT) 25
- Partial Least Squares (PLS) model 25
  - Paal-Knorr reaction with one underlying reaction: 25
  - Paal-Knorr reaction with two underlying reactions: 27

Literaturverzeichnis 29

Toolbox – Details and Tutorials

Introduction

FlowMat is a lightweight, user-friendly MATLAB/Simulink toolbox designed to simplify the modeling, simulation, and optimization of flow reactor systems. With its modular architecture, users can easily replicate real-world setups in MATLAB/Simulink using an intuitive drag-and-drop interface.

The toolbox features a diverse array of reactor components, supporting multiple modeling approaches for tubes and reactors. Users can opt for physically based methods, data-driven techniques, or a hybrid of the two. This flexibility allows seamless integration of various modeling strategies within a single system, ensuring that each reactor component is modeled using the most suitable approach.

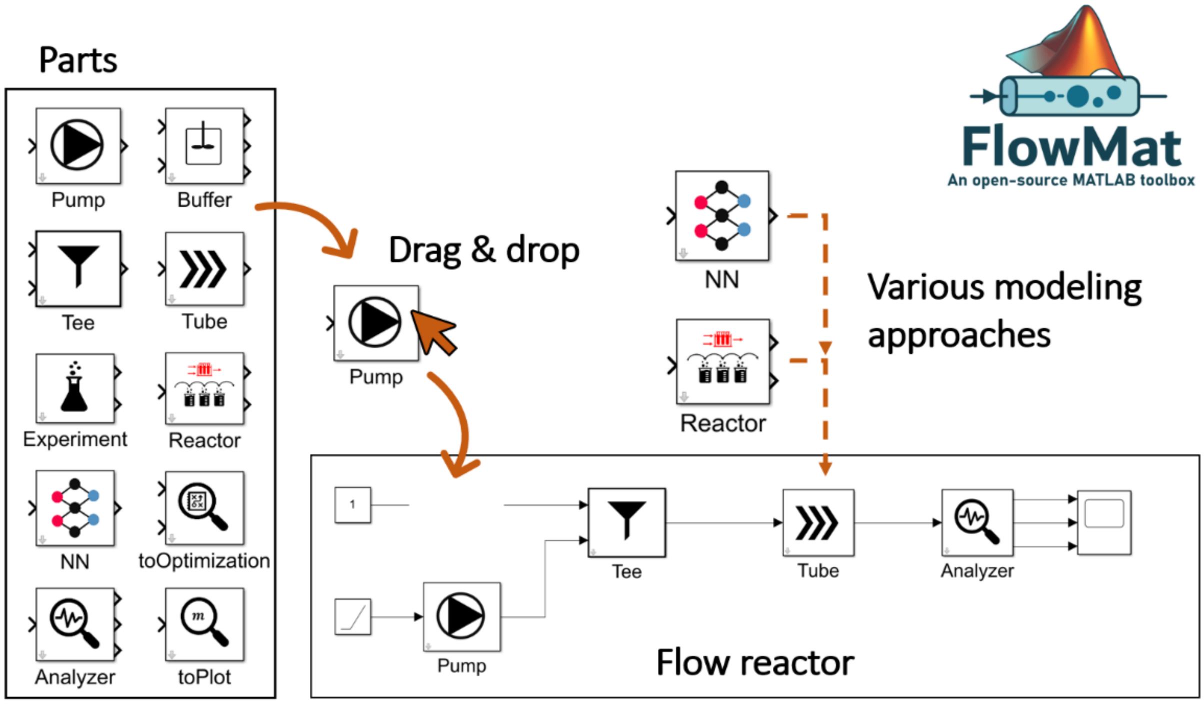

Figure 1: Overview of FlowMat.

Availability

FlowMat is freely available for download and use on GitHub [1], providing easy access for researchers, engineers, and developers to explore its capabilities.

Quantities and units

FlowMat supports commonly used symbols and units, ensuring compatibility with standard conventions. The most relevant quantities, along with their corresponding units, are listed in Table 1.

Table 1: Quantities and units used within FlowMat.

Quantity	Symbol	Unit
Flow rate	q	ml/min
Temperature	T	°C
Molar volume / Subst. conc.	Vm or subConc	mol/ml or mol/L
Tube length	L	m
Discretization time	Td	s
Dispersion coefficient	E or D	m^2/s

FlowMat State

FlowMat employs a unified global interface across all units and components, known as the 'FlowMat State'. This interface encapsulates all the essential information required for seamless communication between units. Only physically relevant quantities are transferred through this interface, ensuring efficiency and clarity. A comprehensive list of all parameters included in the 'FlowMat State' is provided in Table 2.

Table 2: States and according units of a 'FlowMat State'.

State	Unit	Comment
Temperature	°C	The temperature of the liquid (all the species)
flowRates[1-6]	ml / min	An array of flow rates for all 6 species in ml/min. The species are referred to as species A, B, untl F
sub.Conc.[1-6]	mol/L	The molar volume (also referred to as substance concentration) for all 6 species

The 'FlowMat State' facilitates the transfer of key parameters, including temperature, flow rates (in mL/min), and substance concentrations (in mol/L) for up to six species. This default limit can be expanded to accommodate any number of species as needed. The six species are labeled sequentially as species A through F.

All components within FlowMat adhere to this species definition. For certain components, users can explicitly specify which species are affected within a block, with references based on this standardized naming convention.

For example, if a pump is configured to transfer a solvent designated as species 1 (equivalent to species A), the pump's settings allow users to specify that it targets species 1. Additional details on configuring components and species can be found in the relevant sections on parts and tutorials.

Experiments

The FlowMat toolbox enables direct utilization of experimental data through a specialized container known as the ‘experiment-struct’ or simply ‘experiment.’ This struct is designed to store all relevant experimental information in a structured format. To ensure compatibility with the FlowMat toolbox, the experiment-struct must include predefined fields, as these may be referenced during reactor model simulations. A detailed list of the required fields can be found in Table 3. Additional fields can also be appended to the experiment-struct to include supplementary information.

The toolbox includes several predefined experiments (see Table 4) that can be used for testing and verification purposes. Users wishing to incorporate their own experimental data must import the recorded data into the FlowMat toolbox, ensuring that each experiment contains at least the mandatory fields specified in Table 3.

Table 3: Required struct-fields for an 'Experiment-struct'.

**Field / Function**	**Required**	**Info / Comment**
time	all experiments	Time vector of the experiment in s
plot(figId)	all experiments	Plots the experiment in the appropriate figure
TS.totalFlowRate	all experiments	Timeseries of total flow rate in ml/min
TS.flowRates	all experiments	The flow rates of each species in ml/min
sub.Conc.[1-6]	all experiments	The molar volume (also referred to as substance concentration) for all 6 species
TS.temperature	all experiments	Timeseries of temperature
TS.Cin	tracer experiments	Timeseries of the concentrations flowing in the reactor. Thereby, species 1 must be the solvent
TS.Cout	all experiments	Timeseries of the concentrations flowing out of a reactor/tube
TS.Cout<N>	tracer experiments	Timeseries of the concentrations flowing out of segment <N>

Table 4: Available experiments.

**Name / ID**	**Recorded**	**Comment**
Tracer experiment 1 - 9	Williams J., Knoll S. 2024	Experiments with a tracer in a solvent flowing through a setup with three different segments. For some setups, buffers are included. Different delay and dispersion effects for the different segment combinations are recorded
Paal Knorr Reaction – Ramp / Steady state	Silber K. 2024	Paal Knorr Reaction, which forms two products, whereby

		experiments for ramped flow rates and flow rates to reach steady state each time are available
Reduced Paal-Knorr Reaction	Silber K. 2024	Reduced Paal-Knorr Reaction, which forms one product

Parts

Table 5 provides an overview of all available components within FlowMat. In addition to these specialized parts, users can utilize standard Simulink blocks, such as the ‘Scope’ block for visualizing traces or ‘Source’ and ‘Constant’ blocks for defining flow rates.

The table includes a brief description of each block, along with details about the required inputs and possible outputs. Additionally, all mask parameters, which allow customization of the block’s properties, are listed. These mask parameters can be accessed by double-clicking on the respective block in the Simulink model.

Table 5: Available parts/units of FlowMat.

**Parts**	
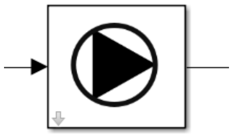<br>Pump	**Pump:**<br><br>The Pump pumps a species with a given/required flow rate q at the input through the system. The output is the globally used FlowMat State and can be connected to any other part of the Toolbox.<br><br>**Input:** Required flow rate q in ml/min<br>**Output:** FlowMat State<br>**Mask:**<br>- Species index ………… Which species is pumped<br>- Temperature in °C …… Temperature of species<br>- Sub. Conc. in ml/L …… Molar volume of species
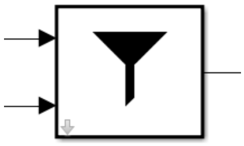<br>Tee	**Tee:**<br><br>Combines two FlowMat States into one FlowMat State by ideally mixing the species, averaging the temperatures, and adjusting the total flow rate accordingly.<br><br>**Inputs:** First and second FlowMat states to be combined<br>**Output:** Combined FlowMat States<br>**Mask:** -
	**Analyzer:**<br><br>Analyze the concentration(s) and total flow rate of a FlowMat State.<br><br>**Inputs:** FlowMat State to analyze

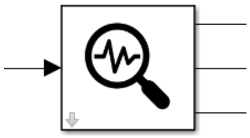<br>Analyzer	**Outputs:**<br>- Total flow rate ml/min<br>- Concentration of species A in mol/L<br>- Concentration of species B in mol/L<br>**Mask:**<br>- Index of species A<br>- Index of species B
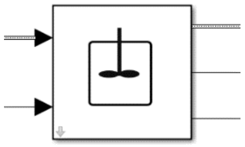	**Tank:**<br><br>A perfectly stirred tank.<br><br>**Inputs:**<br>- FlowMat State flowing into the tank<br>- Flow rate flowing out of the reactor in ml/min<br>**Outputs:**<br>- FlowMat State flowing out of the tank<br>- Total volume in the tank in ml<br>- Fill level in percent<br>**Mask:**<br>➢ **General:**<br>- Sample time in s<br>- Total/Max tank volume in ml<br>➢ **Initial volumes:**<br>- Initial volumes of species 1-6 in ml
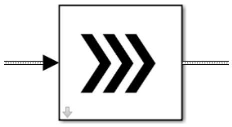<br>Tube	**Tube:**<br><br>Models a tube using transfer functions and a delay element. The transfer function consists of two PT1-elements for which one can define the time constants T1 and T2 in the block mask. Moreover, one can define a delay offset if there are deviations in the theoretical residence time and the measured one. If the characteristic of the dispersion effects changes for different flow rates, 2 more anchor points can be defined, whereby flow rates in between are linearly interpolated.<br><br>**Inputs:** FlowMat State flowing into the tube<br>**Output:** FlowMat State flowing out of the tube<br>**Mask:**<br>➢ **General:**<br>- Tube length in m<br>- Tube diameter in mm<br>- Internal sampling time in s<br>- Maximal delay in s<br>- Tube prefilled with species<br>➢ **Reference point:**<br>- Ref. flow rate<br>- Ref. time constant T1<br>- Ref. time constant T2<br>- Ref. delay offset

<table>
<tr>
<td>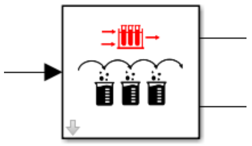
Reactor</td>
<td>Reactor:

Models a flow reactor with a discretized axial dispersion model or the tank in-series model if the dispersion coefficient E is set to zero. Moreover, up to two reactions can be added. Thereby, the reaction can be described by the Arrhenius equation of the form

Ca + Cb -> Cc and
r = Ca*Cb*A*exp(-Ea / (R * T)).

Inputs: FlowMat State flowing into the reactor
Output: FlowMat State flowing out of the reactor
Mask:
➢ General:
- Tube length in m
- Tube diameter in mm
- TiS flow rate factor
- Dispersion coefficient E in m^2/s
- Number of tanks
- Number of species
- Discretization time in s
- Tube prefilled with species
- Maximal delay in s
- Include nominal delay
- Nominal delay offset in s
➢ Reaction 1/2:
- Enable reaction
- Species index Ca
- Species index Cb
- Species index Cc
- Activation energy Ea
- Pre-exponential factor A
- Consumption factor Ca
- Consumption factor Cb</td>
</tr>
<tr>
<td>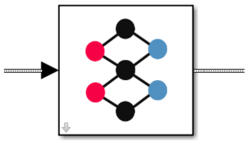
NN</td>
<td>Neural Network:

A Neural Network for Simulink Reactor Modeling: The neural network can be trained on experimental data to replicate delay and dispersion effects or model the entire reactor, including chemical reactions.

Inputs: FlowMat State flowing into the reactor/tube
Outputs: FlowMat State flowing out of the reactor/tube
Mask:
- Neural network ID
- Neural network simulation information
- Sample time Td in s
- Tube prefilled with species</td>
</tr>
</table>

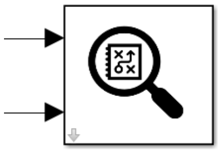<br>toOptimization	**toOptimization:**<br><br>Exports the measured and predicted concentration information to the optimization task. Use this block if you want to optimize the reactor setup, the parameters, or the operation point.<br><br>**Inputs:** FlowMat State before and after reactor(s)<br>**Output:** -<br>**Mask:** -
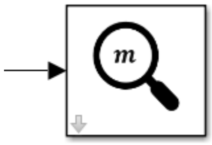<br>toPlot	**toPlot:**<br><br>Exports the space-time information of a reactor system to the Matlab Workspace. The method “FlowMat.plotSpaceTime” can use this information to plot the space-time data of the reactor.<br><br>**Inputs:** Space-time information (xk) of a reactor<br>**Output:** -<br>**Mask:** -
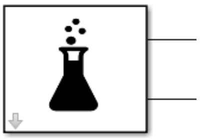<br>Experiment	**Experiment:**<br><br>Exports the space-time information of a reactor system to the Matlab Workspace. The method “FlowMat.plotSpaceTime” can use this information to plot the space-time data of the reactor.<br><br>**Inputs:** -<br>**Output:**<br>- FlowMat State representing the state before the reactor<br>- FlowMat State representing the state after the reactor<br><br>**Mask:**<br>- Experiment

Examples / Tutorial

MATLAB/Simulink

For the FlowMat toolbox, the software solutions MATLAB and Simulink are required. Detailed information about the software itself and how to use it can be found in [2]. For this tutorial, we will not give a detailed explanation of MATLAB/Simulink itself, yet we will mention the most important tools and functionalities to use the FlowMat Toolbox.

FlowMat

FlowMat is an open-source toolbox available on GitHub. To get started, all folders and files can be placed in MATLAB's main working directory. Initializing FlowMat in a MATLAB script is straightforward and requires just a few steps.

For example, in the script

    ‘Example0_HelloFlowMat.m

we provide a simple setup that initializes the toolbox. Once the script is executed, all FlowMat functions and blocks become available for use. After initialization, the MATLAB interface will appear as shown in Figure 2.

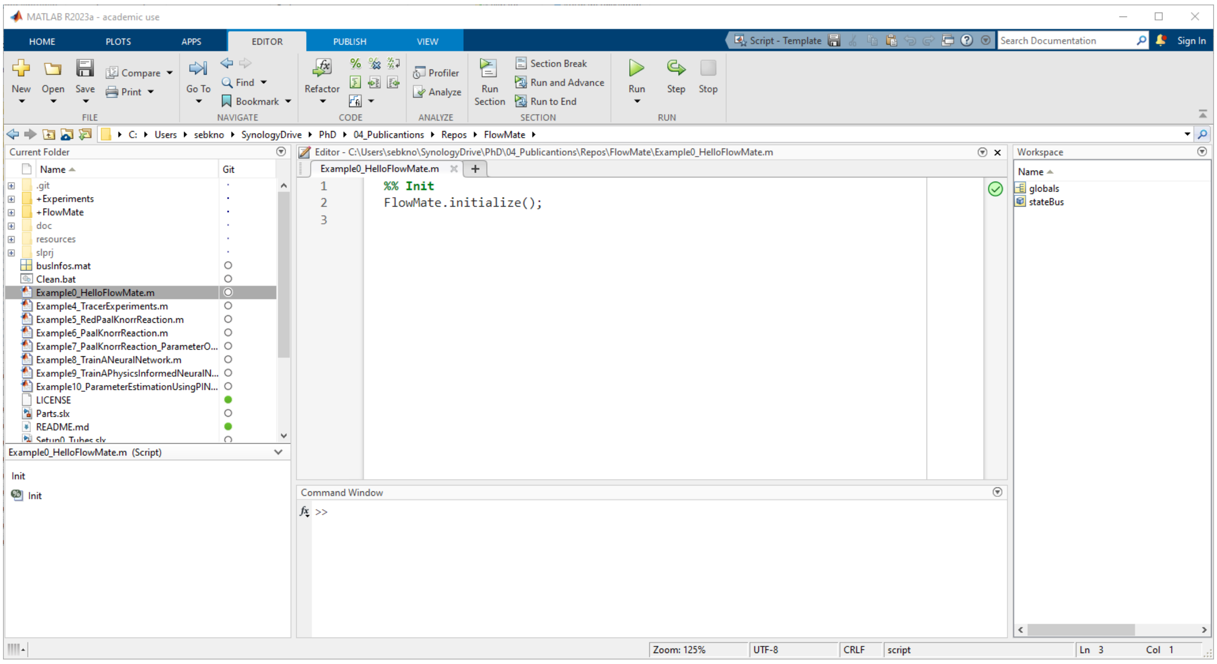

Figure 2: MATLAB and FlowMat.

The figure illustrates the MATLAB interface, with the working directory displayed on the left, the workspace on the right, and the MATLAB script (‘Example0_HelloFlowMat.m’) open in the center. The FlowMat toolbox supports both MATLAB scripts and Simulink models.

To create a Simulink model, navigate to Home > New > Simulink Model. All available Simulink components can be found in Parts.slx, from which they can be copied and used in any reactor project.

Setup with two tubes:

In

    ‘Setup0_Tubes.slx’

we created the first example. In the example, we rebuilt a simple flow reactor, which consists of two tubes, whereby a solute with a dissolved tracer flows through the reactor system. The Simulink model is depicted in Figure 3. The concentration of a tracer in the solvent is measured before and after each segment. When opening the mask of a tube, one can set the tube length, diameter, but also the time constants of the PT1-element for various anchor points. After initializing FlowMat with

```
FlowMat.initialize();
```

in a MATLAB script, one can start the simulation. After the simulation finished, the output concentration of the tracer after each tube can be found by double-clicking one of the scope blocks.

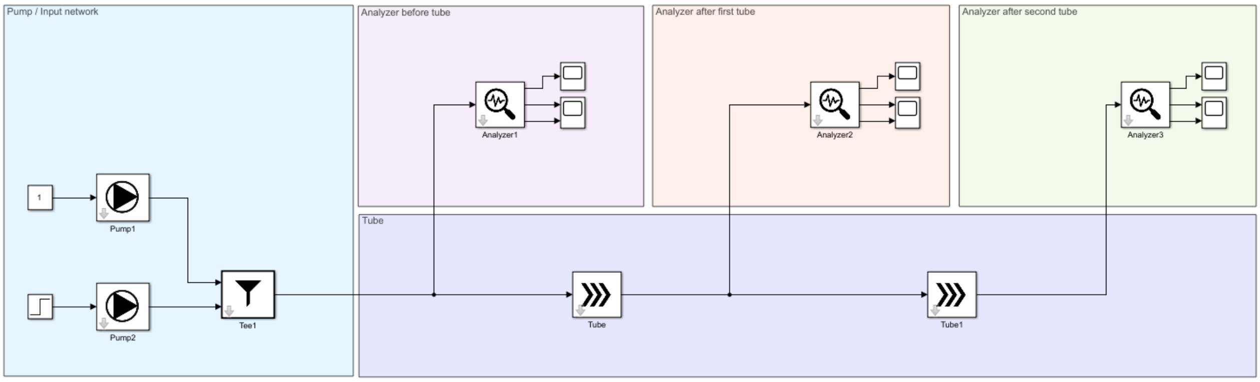

Figure 3: Simulink model of a flow reactor with two tubes.

Setup with an Axial dispersion model / Tank in Series model:

In the

`‘Setup1_ADTiSModel.slx’`

we created a flow reactor setup as depicted in Figure 4. Thereby, we model a flow reactor with an AD/TiS model. The AD/TiS modeling approach can also be used to model delay and dispersion effects, whereby the dispersion effects are more physically motivated. Moreover, this modeling approach can be used to include reactions, which we will do in the next example. By disabling all reactions in this setup and defining a tube length and all other meta parameters, we again get a reactor setup, which can be used to simulate simple delay and dispersion effects.

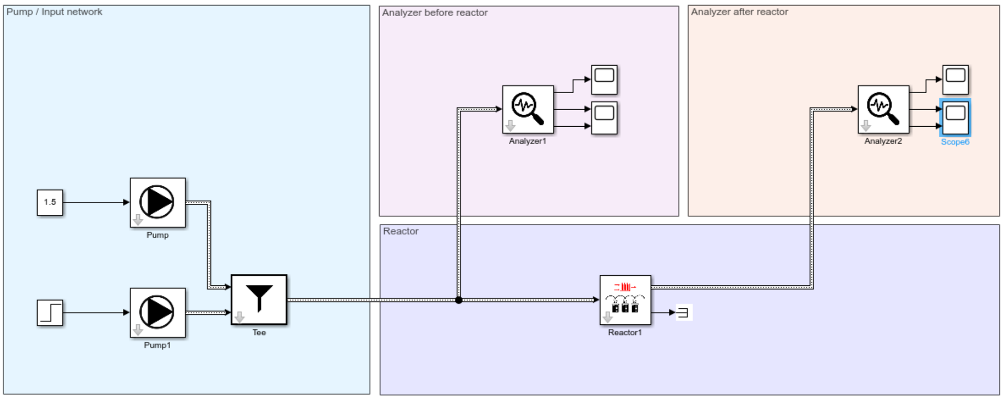

Figure 4: Example setup with an AD/TiS model to simulate a simple flow reactor.

Setup with a stirred tank:

In the

`‘Setup2_AFlowReactorWithAStirredTank.slx’`

we created a setup with a tube and a stirred tank. For the setup, the considerations for the tube are similar to those before. For the flow rates of the pumps, we kept the flow rate of species A (for instance, a solvent) constant, and the flow rate of species B (for instance, a tracer) oscillated between zero and 1 ml/min. Thus, an oscillating concentration of species 2 (the tracer) is the result after the tee and the tube.

The stirred tank was placed after the tube, and within its mask, the properties and initial conditions are defined accordingly. After simulating this setup, one can see that the oscillating concentration of species B in front of the stirred tank is damped out after the tank.

Note that the flow rate out of the tank is changed during the experiment. Thus, the species level within the tank rises in the beginning as more liquid flows into the tank and decreases after increasing the flow rate.

Setup with an AD/TiS model with reaction:

In the

`‘Setup3_AFlowReactorWithReaction.slx’`

we created a setup to rebuild a simple reactor with a reaction. Thereby, two species are pumped into a tube where they first experience delay and dispersion effects in the first section. For this first section, we use a simple tube model as we assume that there is no reaction happening. Our considerations for defining the meta-parameters of the tube are similar to those before.

After the tube, there is a section where the two species are reacting and forming a third species. Thus, we use the AD/TiS model for this section, which allows us to include a reaction. Within the mask of the AS/TiS model, we can enable the first reaction and specify the parameters of the underlying reaction. Thereby, the reaction is described by the Arrhenius equation, and quantities like the activation energy and pre-exponential factors have to be defined. Moreover, we can define the proper indices of the species as species 1 (species A) + species 2 (species B) form species 3 (species C).

The setup can be found in Figure 5 and the corresponding settings for the AD/TiS model in Figure 6.

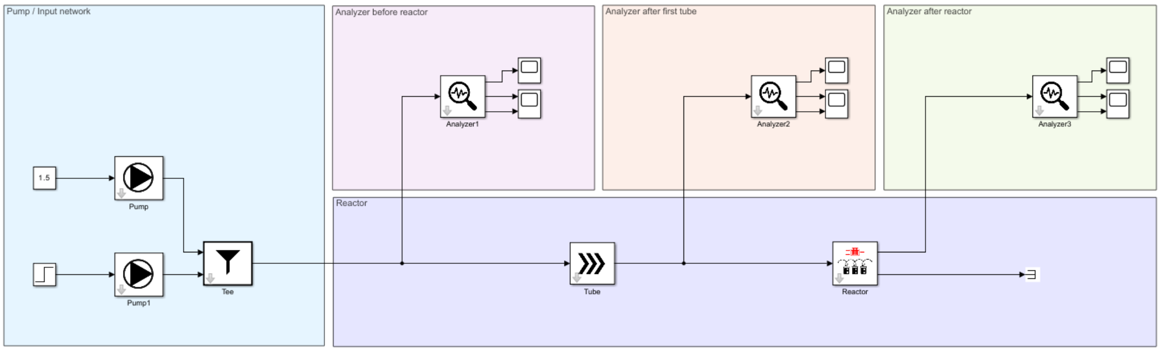

Figure 5: Example setup with two parts whereby the second segment includes a reaction.

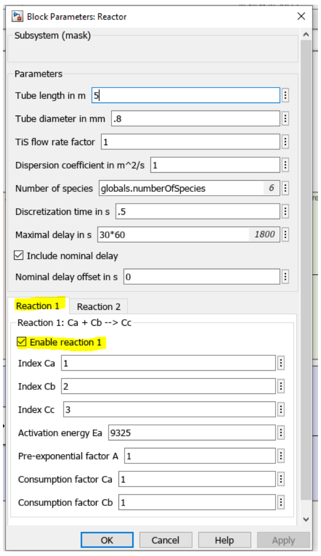

Figure 6: Mask settings for the AD/TiS model to include a reaction.

Tracer Experiments

We recorded several tracer experiments for different setups in a real-world reactor setup. Thereby, a tracer experiment consists of a tracer within a solvent flowing through the reactor setup. The reactor setup is comprised of three segments, whereby the concentration is measured between each of those segments. A segment is a tube that is varied in diameter and length within the different experiments. Thus, we face different dispersion and delay effects throughout the experiments. The reactor setup itself is depicted in Figure 7.

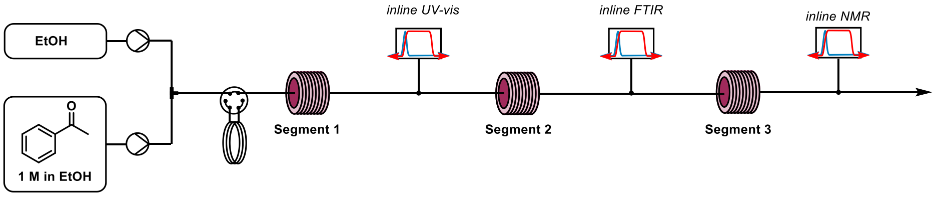

Figure 7: Reactor setup for tracer experiments with three segments and three positions where the concentration of the tracer is measured.

In the Simulink-File

```
‘Setup4_TracerExperimentSetup.slx’
```

we created a setup to rebuild the mentioned reactor setup with the three segments and the three analyzers. As we want to compare it with the real-world measurements, we imported the experimental data in the proper FlowMat-Experiment format as described in the section ‘Experiments’. Thus, it is possible to use an ‘Experiment’-block to directly use the measurement data within the Simulink model and connect it to the same ‘scope’-block as the predicted ones to directly validate them. The according Simulink-File and the rebuilt setup are depicted in Figure 8. Before we can make use of the ‘Experiment’-block, we have to load the correct experiment into the workspace. We can do this by writing:

```
experiment = Experiments.loadExperiment('Tracer Experiment 1');
experiment.plot(1);
```

after the FlowMat initialization within our MATLAB script. As there are 9 tracer experiments, we can adopt the number accordingly. One can see that we also make use of the ‘plot’-Method which the experiment-struct provides. This method can be used to validate that we have loaded the correct experiment. One can find all the required lines to run the Simulink file in

```
‘Example4_TracerExperiments.m’.
```

After running the MATLAB script (‘Example4_TracerExperiments.m’) we can start the simulation (in ‘Setup4_TracerExperimentSetup.slx’ by clicking ‘Simulation’ > ‘Run’).

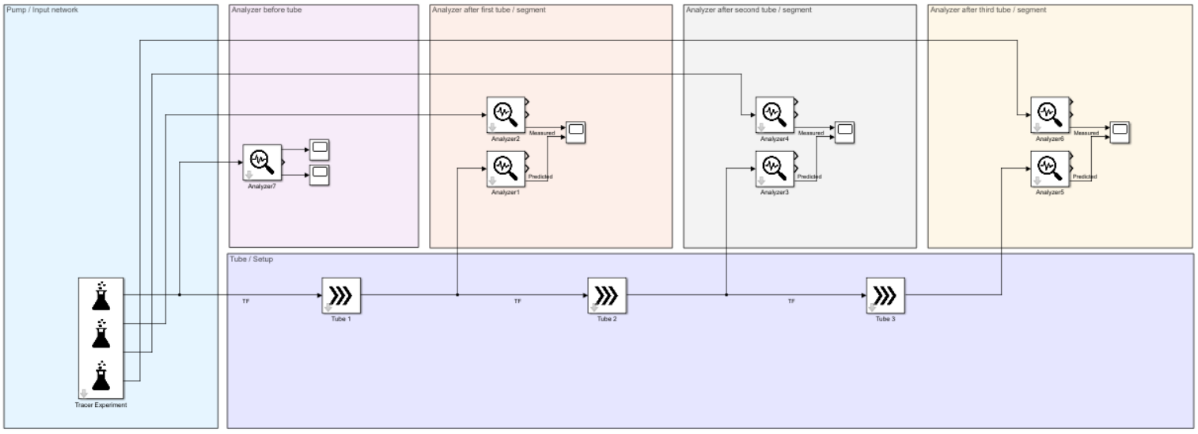

Figure 8: Simulink-File in which we rebuilt the experiment setup for the tracer experiments with three different segments and the analyzers in between each segment.

When adjusting the tube settings in the ‘Tube ’ block (double-clicking) in such a way that they match the values from the real-world experiment (length and diameter), we should see a comparable trace in the ‘scope’ block when it comes to the delay. For adjusting the dispersion effects, one can tune the time

constants T1 and T2 of the PT1-elements. There might be a change in this dispersion effect throughout different flow rates. If this is the case, one can make use of two more anchor points whereby values between two flow rate reference points are interpolated as described before. Moreover, one can define a delay offset if the theoretical delay does not match perfectly with the measured one. Here, it is also possible to vary this delay offset throughout the different reference points.

We did the adjustment of the tube settings for the first experiment in the 'Setup4_TracerExperimentSetup.slx' accordingly. So, one should see similar traces as depicted in Figure 9 after running the simulation.

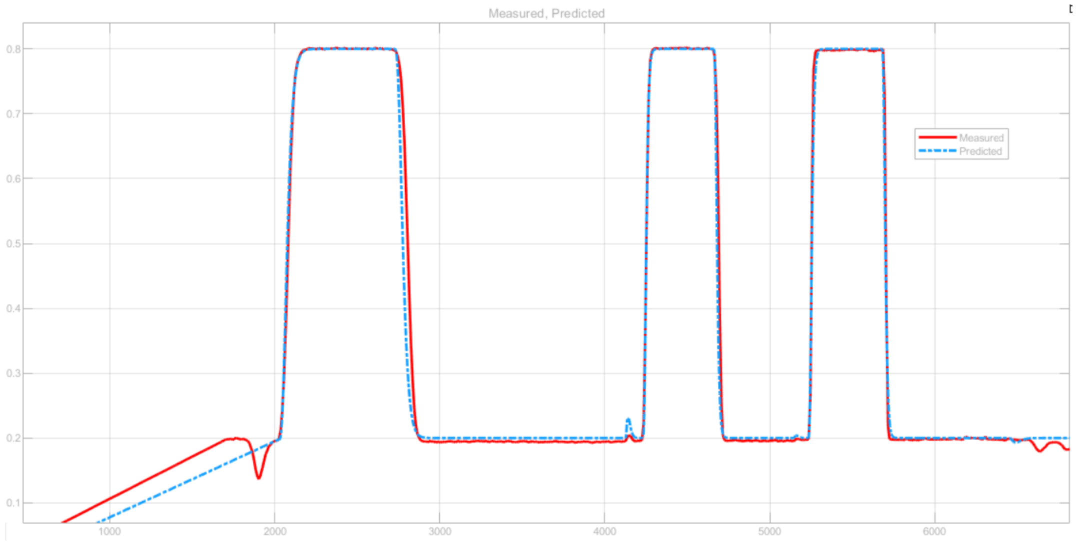

Figure 9: Predicted and measured traces of the tracer concentration after the second segment.

One can do the same for all other tracer experiments by adjusting the tube parameters to match those of the tubes that were used in the real-world experiment. If you have a segment where you already have identified the tube parameters (As the same tube might be used in the same experiment but maybe within a different segment), you can use the already identified parameters.

Reduced Paal-Knorr Reaction

We also recorded experiments with reactions taking place in the reactor. The first set of experiments incorporates the reduced Paal-Knorr Reaction. For this reaction, two species react to form a third one. In the MATLAB script

```
'Example5_RedPaalKnorrReaction.m'
```

we initialize the FlowMat Toolbox and load the experiment similar to the example with the tracer experiments. Moreover, we start the simulation already in the MATLAB script with the 'sim'-Method. Thereby, we simulate the Simulink file

```
‘Setup5_RedPaalKnorrReaction_ReactorSetup.slx’
```

in which we rebuilt the real-world reactor setup.

The Simulink file makes use again of the ‘Experiment’ block and ‘Analyzer’-blocks to directly compare the measurements with the predictions. As a reaction takes place within the tube/reactor, we make use of the ‘Reactor’ block, which uses a discretized axial dispersion model or the tank in series model to incorporate the reactions. All relevant tube and reaction parameters can be defined in the mask of the ‘Reactor’ block. Additionally, we make use of the ‘toPlot ’ block such that we can plot the space-time information within the reactor afterwards. The resulting Simulink files look as depicted in Figure 10.

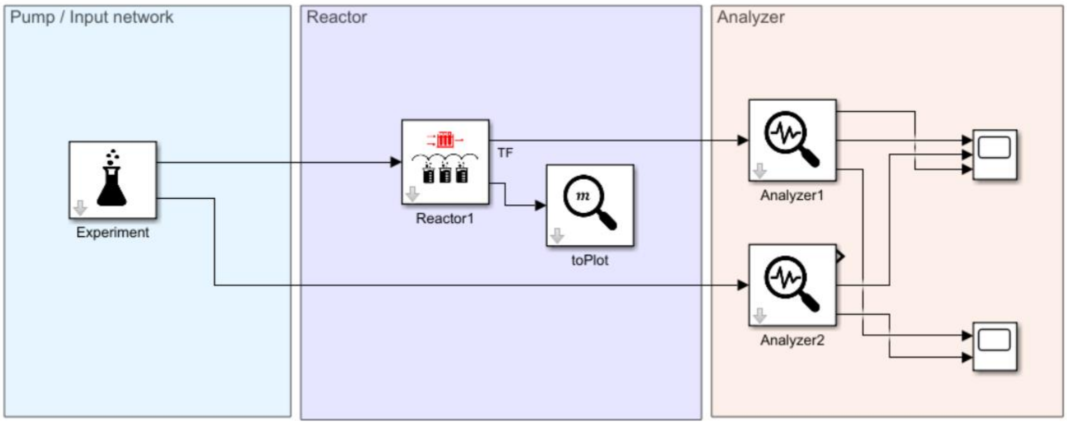

Figure 10: Simulink-file for the reactor setup with reaction.

After simulating the reactor setup within the MATLAB script, we can make use of the ‘plotSpaceTime’-method and the ‘plotComaprison’-method. The script might be extended by the following lines

```
FlowMat.plotSpaceTime(simResult, [2, 3, 4], ["Ethanol", "Hexa",
"Prodcut"])
FlowMat.plotComparison(experiment, simResult, [3, 4])
```

which plots the space-time information of the reactor and the comparison with the measurements of the real-world experiments.

Here, it is possible to define which species indices should be plotted and how they should be labeled. In the example, this is done with the ‘plotSpaceTime’ method by defining the second parameter as an array of [2, 3, 4] to define that only species 2 (= species B), species 3 (= species C), and species 4 (= species D) should be plotted. With the third parameter, the labels of those species are defined accordingly. Thus, the resulting plot will look as depicted in Figure 11.

Similarly, the species to be compared are defined as the third parameter in the ‘plotComparison’-method. Here, it would also be possible to define labels

within the fourth parameter. Yet this is not required. The comparison plot looks as depicted in Figure 12.

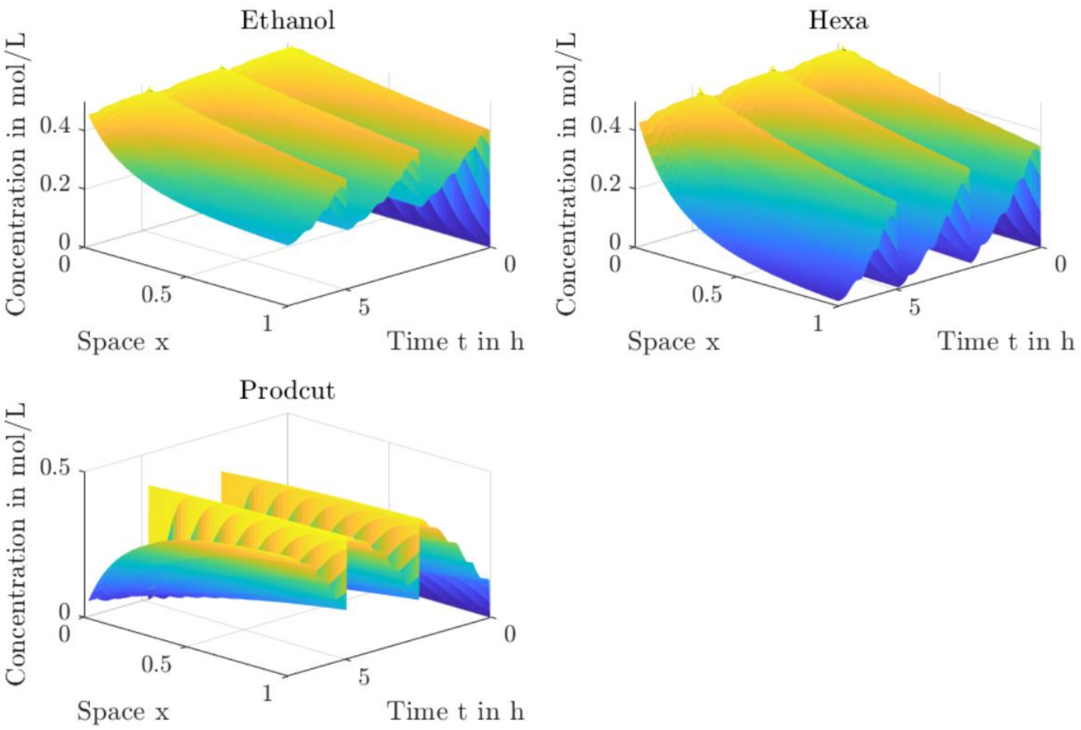

Figure 11: Space-time results of the simulation of the reactor setup with reaction.

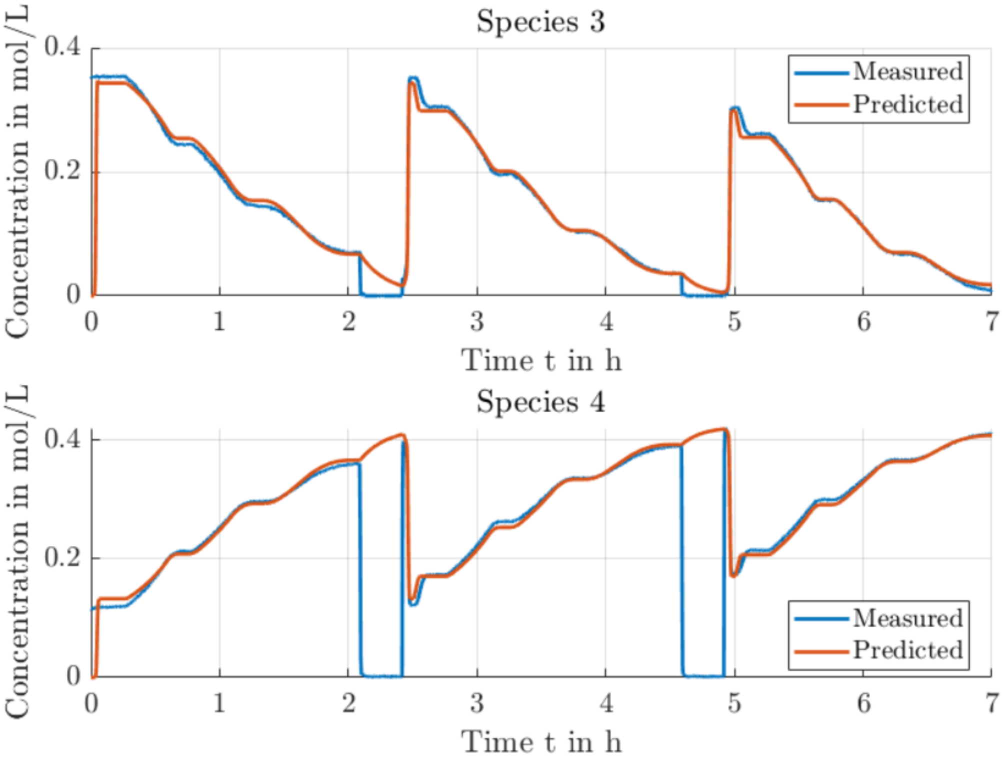

Figure 12: Comparison plot of the measured traces and the simulated traces.

Paal-Knorr Reaction

We also recorded experiments with two underlying reactions. For the Paal-Knorr Reaction, we have two species that form an intermediate product. This intermediate product reacts with one of the starting species to form the final product.

In the MATLAB script

```
'Example6_PaalKnorrReaction.m'
```

we load the experimental data and simulate the 'Seup6_PaalKnorrReaction_ReactorSetup.slx' within the MATLAB script. The MATLAB script and the Simulink file are very similar to the setup with the reduced Paal Knorr Reaction. Only the parameters and settings within the 'Reactor' block are changed. Here, the second reaction is enabled, and the parameters from external sources are used.

After running the MATLAB script ('Example6_PaalKnorrReaction'), one will end up with a space-time plot and a comparison plot as depicted in Figure 13 and Figure 14, respectively.

For the comparison plot, one can see that the prediction could be slightly improved. This can be achieved by tuning the reaction parameters or using an optimization. How a parameter optimization can look will be covered in the next example.

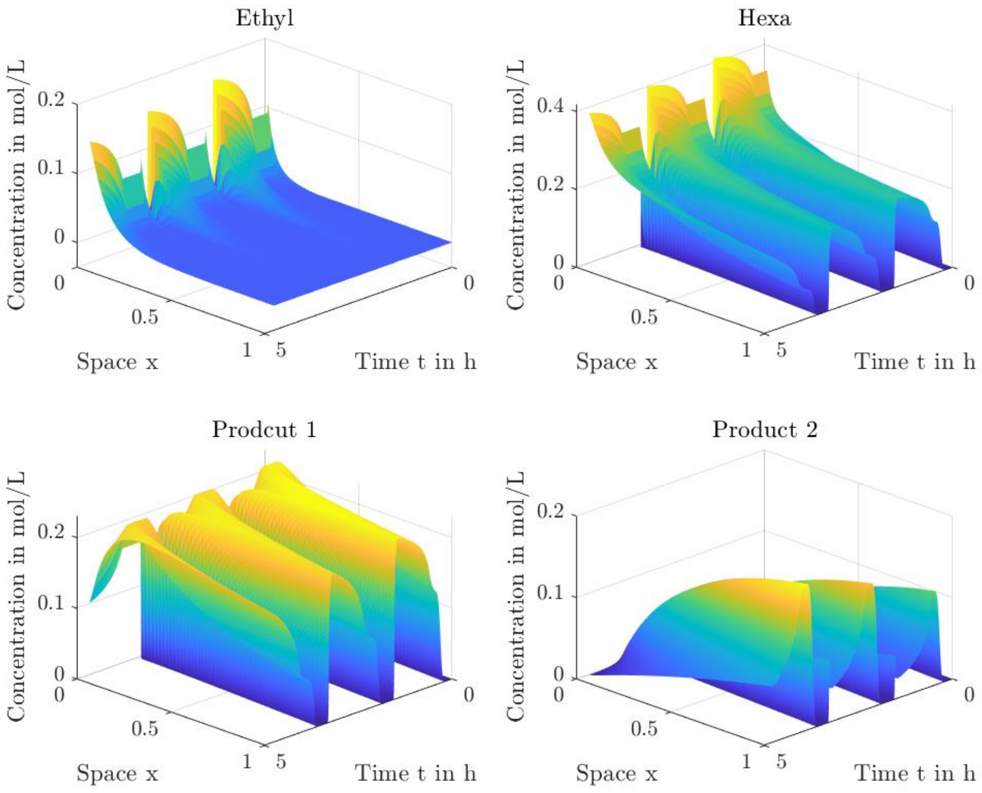

Figure 13: Space-time results of the simulation of the reactor setup for the Paal Knorr Reaction.

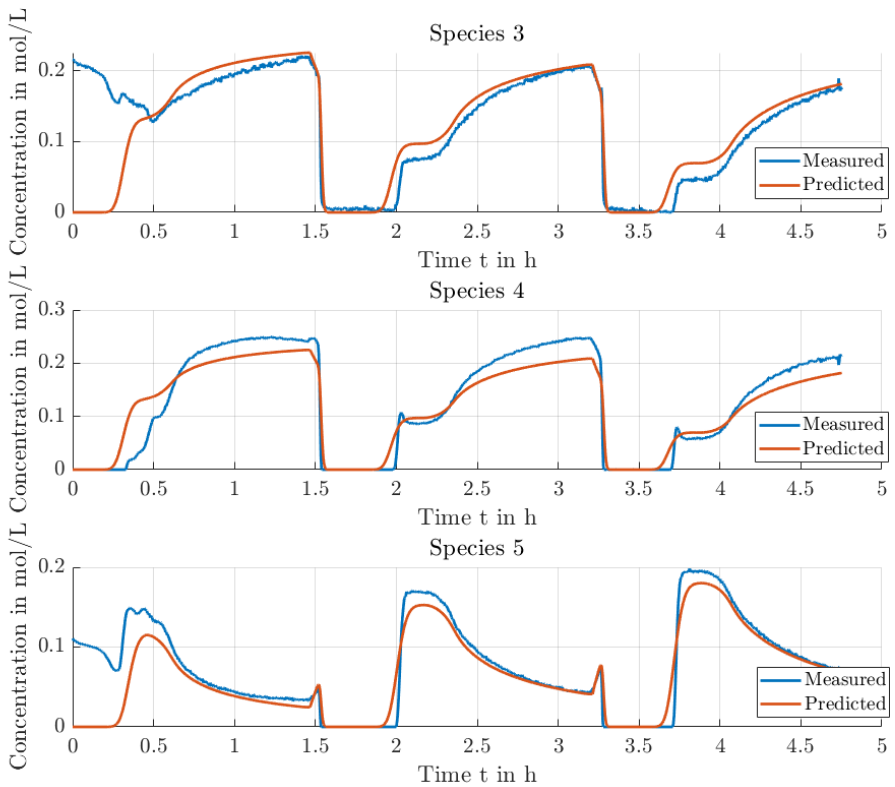

Figure 14: Comparison plot of the measured traces and the simulated traces for the Paal Knorr Reaction.

Paal-Knorr Reaction – Parameter optimization

As mentioned in the example before, it might be of interest to optimize the reaction parameters to get more accurate simulation results or to get the reaction parameters at all.

Thus, the FlowMat Toolbox offers the method ‘optimization’, which can be used to optimize parameters. In the MATLAB script

‘Example7_PaalKnorrReaction_ParameterEstimation.m’

we created such a template script to optimize the given reaction parameters for the Paal-Knorr reaction. In the script, the experiment is loaded and simulated with the initial estimates for the reaction parameters. Thus, the first simulation gives the same results as the example before. To enable an optimization of the reaction parameters, we defined extra variables for the reaction parameters in the MATLAB script. This extra variable can use within the Simulink file (‘Setup6_PaalKnorrReaction_ReactorSetup_Optimization.slx’). In the mask of the ‘Reactor’ block, the variables are used instead of exact values.

When it comes to the optimization itself, we have to define the starting parameters. Moreover, we can define the upper and lower bounds of those parameters. If there are no bounds, one can set the upper or lower bound to be an empty array or to set it to a large enough number. In the MATLAB script, this might look like

```
x0 = [A1,    A2,      Ea1,     Ea2];
```

```
lb = [0.1,  0.1,    1e3,    1e3];
ub = [10,   10,     1e5,    1e5];
```

Furthermore, we have to define the variable names as an array of strings, such that the underlying optimization method knows how we named the variables that can be optimized. We also have to define the name of the Simulink file in which we rebuilt the reactor setup. As most of the time, not all resulting concentrations are measured, one can define which species the optimization should use for the optimization itself. Additionally, it is possible to define a variable (for instance, 'timeIntervalsOfInterest') that defines which time intervals are of interest and which should be used for the optimization. This is due to the fact that measurements might include some parts which are not representative. For our example, this might look like

```
variableNames = ["A1", "A2", "Ea1", "Ea2"];
simFilename =
"Setup7_PaalKnorrReaction_ReactorSetup_Optimization.slx";
speciesIdxOfInterest = [3, 4]; % Diketon, Product

timeIntervalsOfInterest = [ ... % Time: Min, Max
    2500, 5450; ...             % Interval 1
    7400, 11750; ...            % Interval 2
    13500, 1e6; ...             % Interval 3
];
```

Before we can start the optimization, we have to adopt the Simulink file by adding a 'toOptimization' block. This block is required, such that the optimization has access to the resulting simulation results. The adaptation might look like depicted in Figure 15.

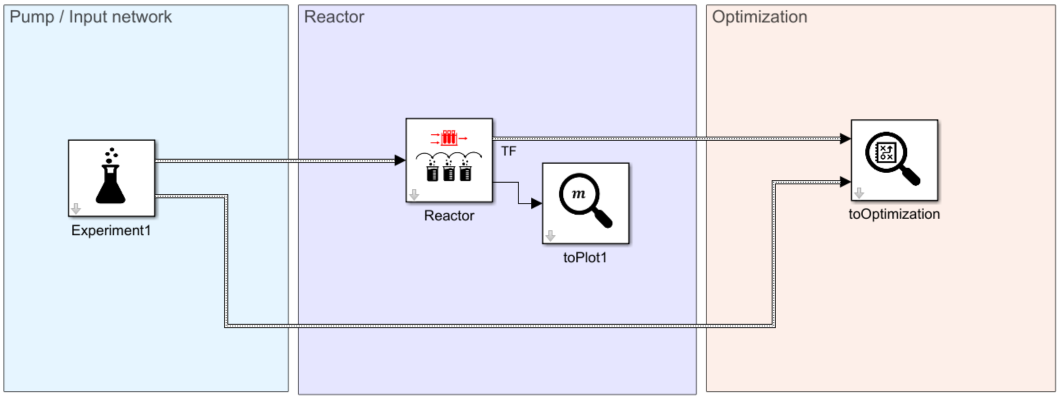

Figure 15: Adaptation of the reactor setup by the 'toOptimization'-block.

Having defined all variables and adopted the Simulink-file, one can start the optimization by using

```
xOpt = FlowMat.optimization(x0, variableNames, simFilename,
  speciesIdxOfInterest, lb, ub, timeIntervalsOfInterest,
                    verificationPlot);
```

It makes use of the defined Simulink file to optimize the defined parameters. To have a visual preview, one can set the parameter ‘verificationPlot’. This results in the intermediate plot, which can be depicted in Figure 16Figure 16. Here, one can also see the defined time intervals and verify them once more.

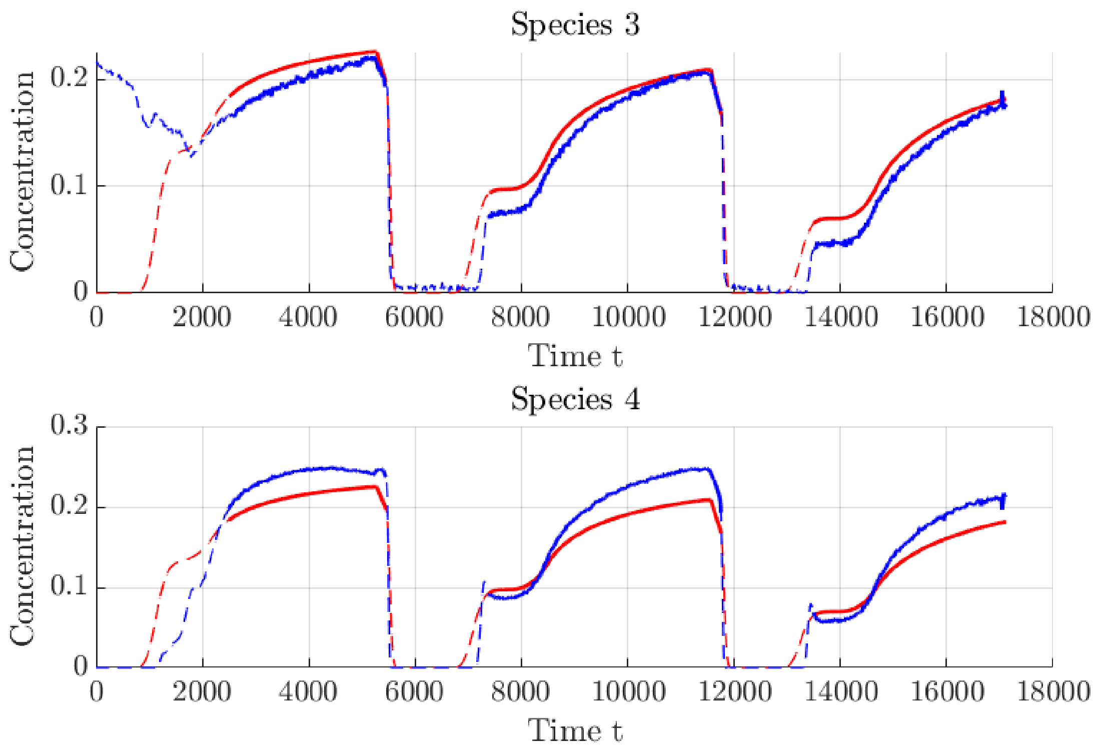

Figure 16: Verification plot during an optimization of reaction parameters.

After the optimization, the ‘optimization’ method returns the optimal reaction parameters within one vector. Those values can be extracted, and the pre-defined parameter variables can be overwritten. In the MATLAB script this might look like

```
A1 = xOpt(1); A2 = xOpt(2);
Ea1 = xOpt(3); Ea2 = xOpt(4);
```

The new values can be used to simulate the setup once more. The final result should deliver more accurate predictions. A simulation with optimized reaction parameters might look like that depicted in Figure 17.

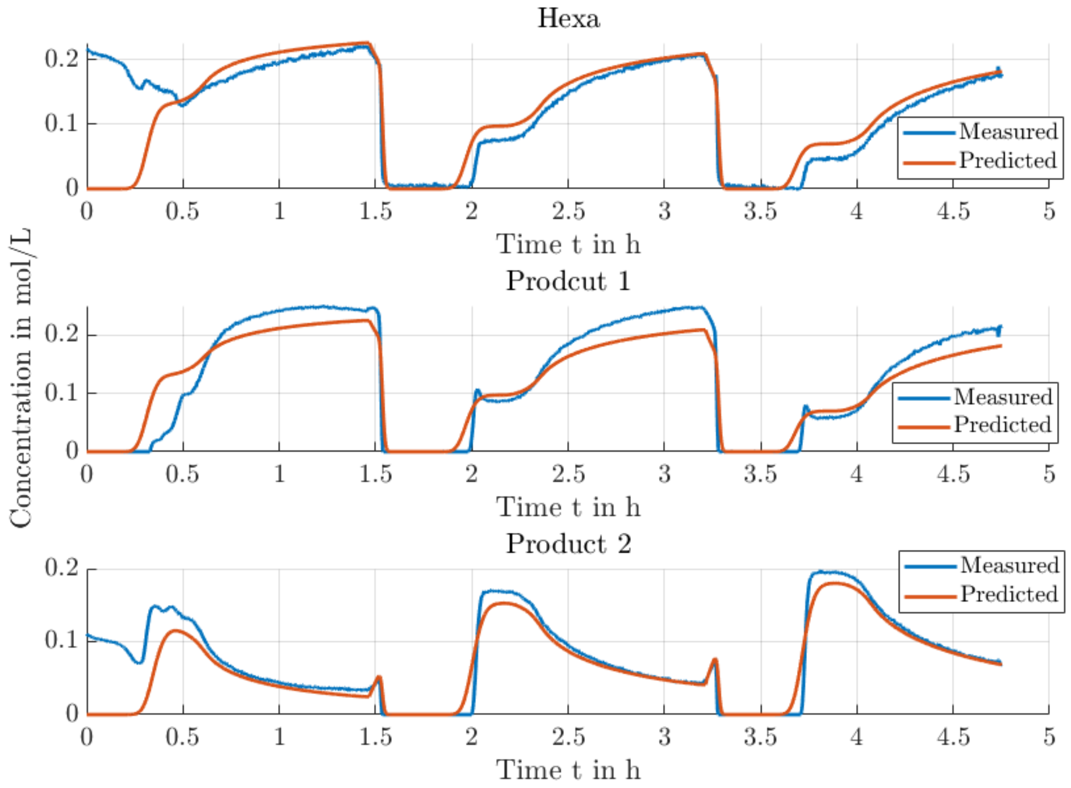

Figure 17: Comparison of simulated and predicted concentrations after optimization of the reaction parameters.

Neural Networks

The FlowMat Toolbox also provides the possibility, to use data-driven models like neural networks. Thereby, the FlowMat Toolbox tries to keep the programming and the designing of the neural network as simple as possible. Thus, there is one simple 'trainNeuralNetwork'-method that can be used to train a shallow multilayer perceptron neural network. The necessary parameters are the experiment itself and the indices of the species that are measured and for which the neural network can be trained. In the MATLAB script this might look like

```
NN1 = FlowMat.trainNeuralNetwork(experiment, [3, 4]);
```

As sometimes more advanced neural network structures are required, one can also define important meta parameters such as the layer structure, number of epochs, etc., with the optional parameters of the method.
Once the neural network is trained, the accuracy of the neural network can be checked with the 'verifyNeuralNetwork'-method. In the MATLAB script, this might look like

```
FlowMat.verifyNeuralNetwork(NN1, experiment);
```

Which results in plot as seen in Figure 18.

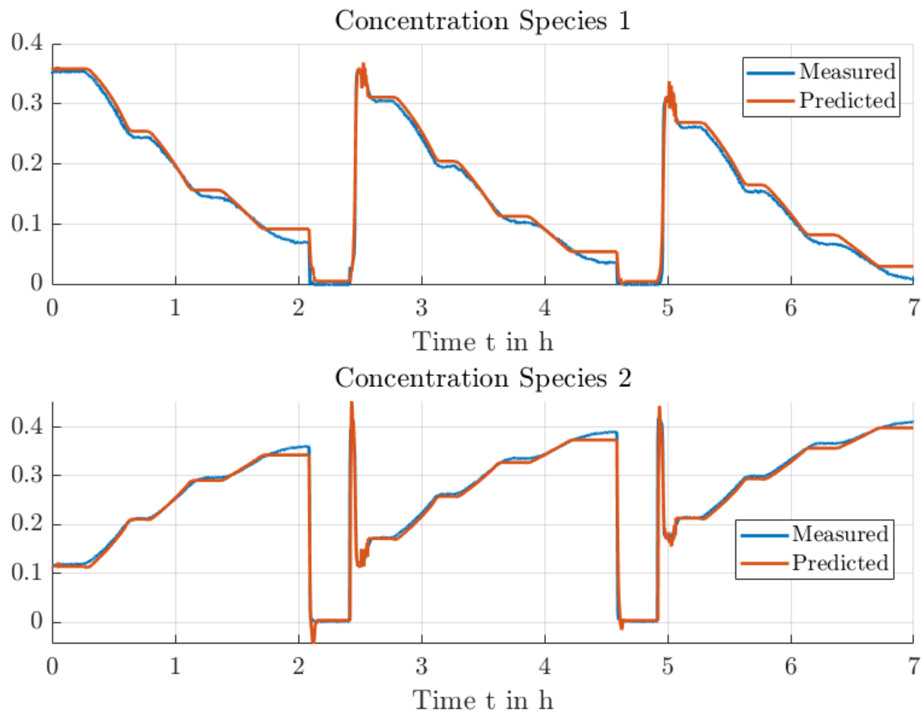

Figure 18: Verification of a neural network using the 'verifyNeuralNetwork'-method.

The resulting neural network can also be used within any Simulink file. Here, the ‘NeuralNetwork’-Block (or ‘NN’-Block) can be used. In the mask of the block, it is important to select the correct ID of the neural network. Here, all neural networks must be named with ‘NN’ in the beginning, followed by an ID. For example, if a neural network is named ‘NN1’, the corresponding ID is 1. In Figure 19, a Simulink file using the ‘NN’-block is depicted.

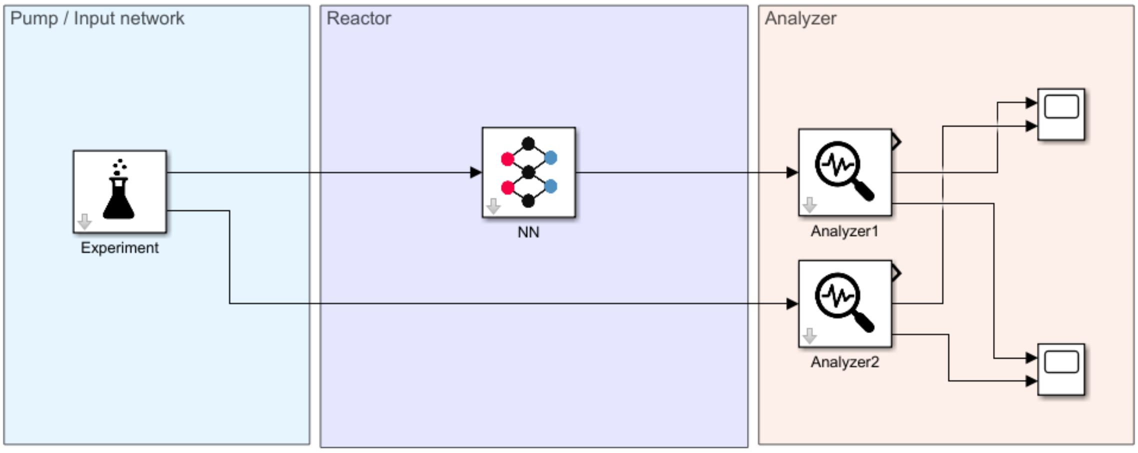

Figure 19: Simulink-file using a 'NN'-block within the setup.

Physics-Informed Neural Networks

In the examples:

- `Example9_TrainAPhysicsInformedNeuralNetwork.m`
- `Example10_ParameterEstimationUsingPINN.m`

we make use of

```
FlowMat.trainPhysicsInformedNeuralNetwork(…)
```

to train a PINN. The first example demonstrates the Paal-Knorr reaction with a single reaction, while the second example extends this to two reactions. Both examples incorporate a custom loss function for training, which is defined within the respective examples. The custom loss allows the integration of physical constraints and models, such as the axial dispersion model. Additionally, users can include extra parameters to be predicted, such as those derived from the integrated physical model.

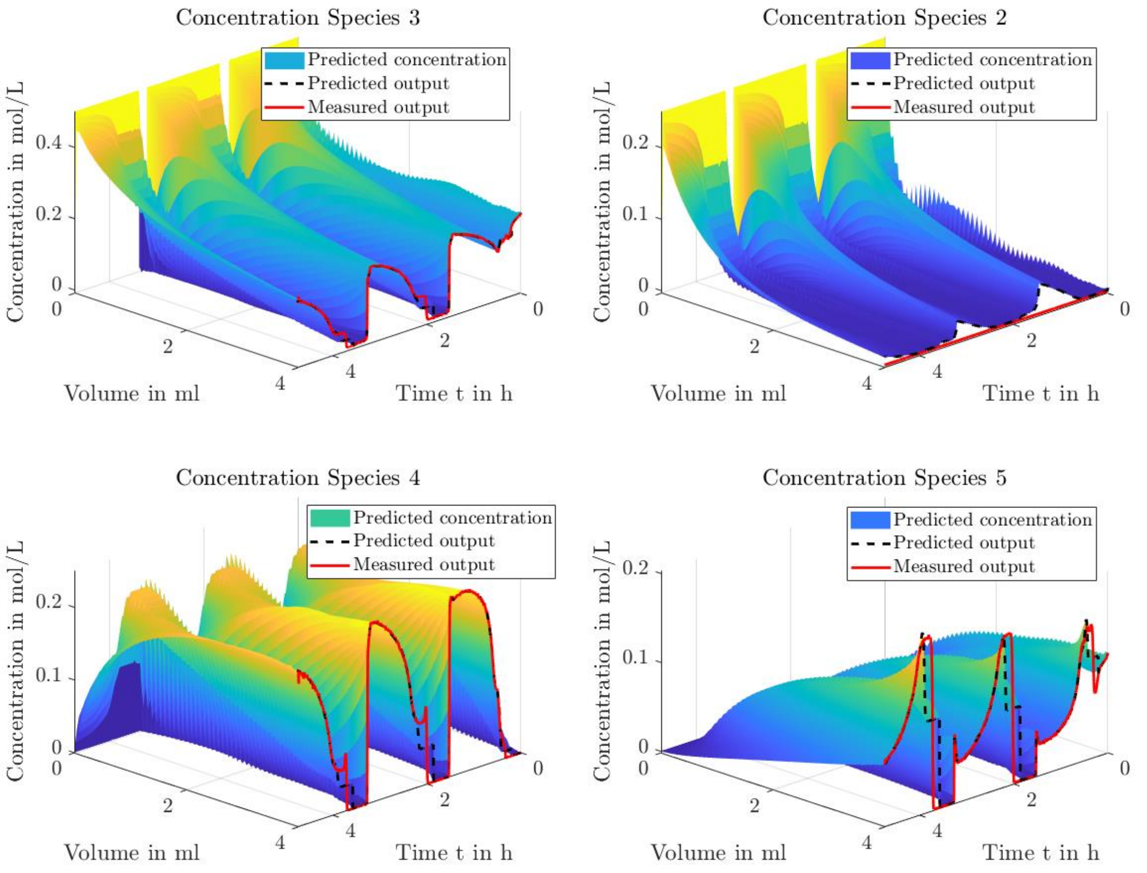

Figure 20: Space-time insight of the final PINN for the Paal-Knorr Reaction with one underlying reaction.

Reactor setup

Reactor

The feed solutions were delivered using Knauer AZURA P 4.1S HPLC pumps (10 mL/min pump head, Hastelloy/ceramic with pressure sensor). A back pressure regulator (BPR, Upchurch, P-465) equipped with a 34 bar (green, P-765) cartridge was attached directly after each HPLC pump. A 7-port mixer was used to combine the inlet streams. The coil reactor (5.00 mL for Paal-Knorr reaction forming one product, 4.20 mL for Paal-Knorr reaction forming two products, PFA tubing, 0.8 mm i.d.) was connected using PFA tubings (0.8 mm i.d.) and heated using a thermostat (Huber, Ministat 240). The reaction mixture was analyzed using inline FTIR (Mettler Toledo, React IR 15) with a flow cell (Mettler Toledo, DS Micro Flow Cell Diamond). A membrane-based BPR (Zaiput, BPR-10), set to 5 bar was integrated after the FTIR.

Automation

The HPLC pumps and the thermostat were connected *via* RS232 to the HiTec Zang LabManager. The ramps for the flow rates and temperature were programmed in HiText (HiTec Zang) and the setpoints for the HPLC pumps and the thermostat were set in LabVision software (HiTec Zang).

Process Analytical Technology (PAT)

Inline FTIR spectra were recorded on a Mettler Toledo ReactIR 15 equipped with a flow cell (Mettler Toledo, DS Micro Flow Cell Diamond). The acquisition time for each data point was 15 s. Spectra were recorded between 4000 and 600 cm^{-1} using the maximum resolution of 4 cm^{-1}. The obtained spectra were exported by the iCIR7 software and automatically read and processed with a PLS model using Peaxact Process Link (S-PACT).

Partial Least Squares (PLS) model

Paal-Knorr reaction with one underlying reaction:

Training samples were obtained from calibration solutions from mixtures of 2,5-hexandione, ethanolamine and product in concentrations ranging from 100-500 mmol/L including blank samples from *iso*-propanol. Each sample was injected to the IR flow cell and at least five spectra were collected for each sample. The acquired training spectra were read into PEAXACT 5.3 (S-PACT) and processed with the following pretreatment conditions. The global range was set to 600-1900 cm^{-1}. A rubber band subtraction was used for baseline correction and the spectra were further processed using 1st derivative with a filter length of 5 for smoothing. A sample spectrum of the reaction mixture before and after pre-processing is shown in Figure 21 and Figure 22Fehler! Verweisquelle konnte nicht gefunden werden.. The following ranks were set for the components in the PLS model with the resulting root-mean-square error of

calibration ($RMSE_C$) and root-mean-square error of cross-validation ($RMSE_{CV}$) as a performance indicator: 2,5-hexandione (rank 8, R^2: 0.9999, $RMSE_C$: 1.15 mM, $RMSE_{CV}$: 2.44 mM), ethanolamine (rank 5, R^2: 0.9995, $RMSE_C$: 4.42 mM, $RMSE_{CV}$: 4.97 mM), product (rank 4, R^2: 0.9999, $RMSE_C$: 1.40 mM, $RMSE_{CV}$: 1.53 mM).

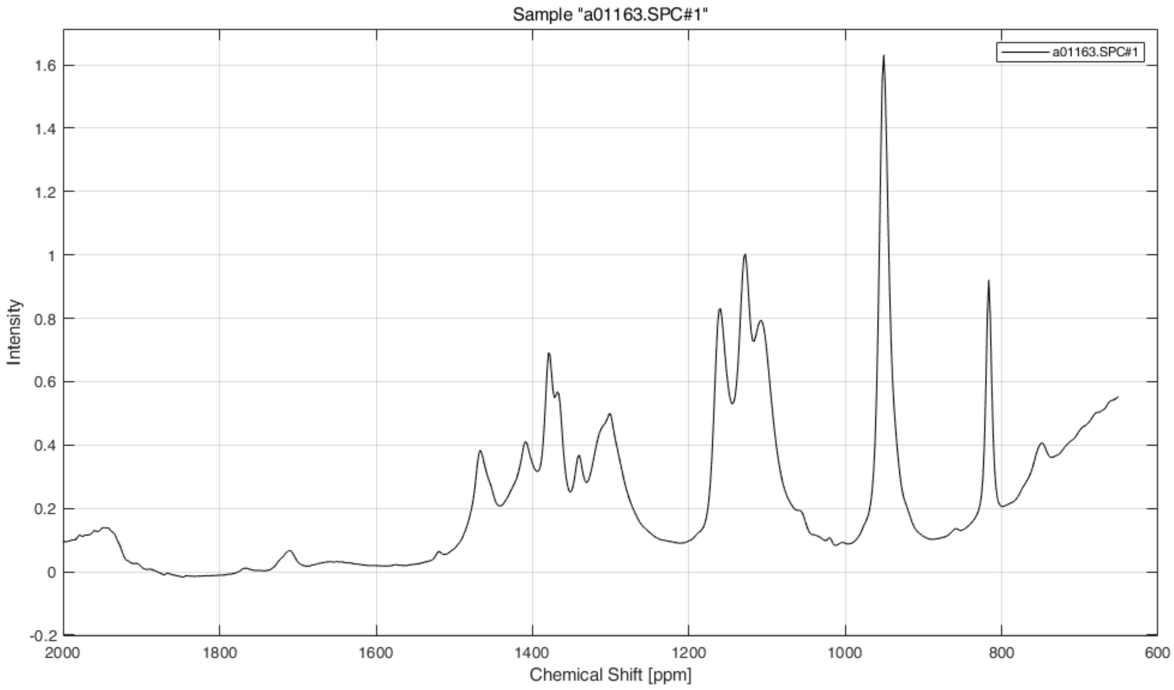

Figure 21: FTIR spectrum of the Paal Knorr reaction mixture forming one product before pretreatment.

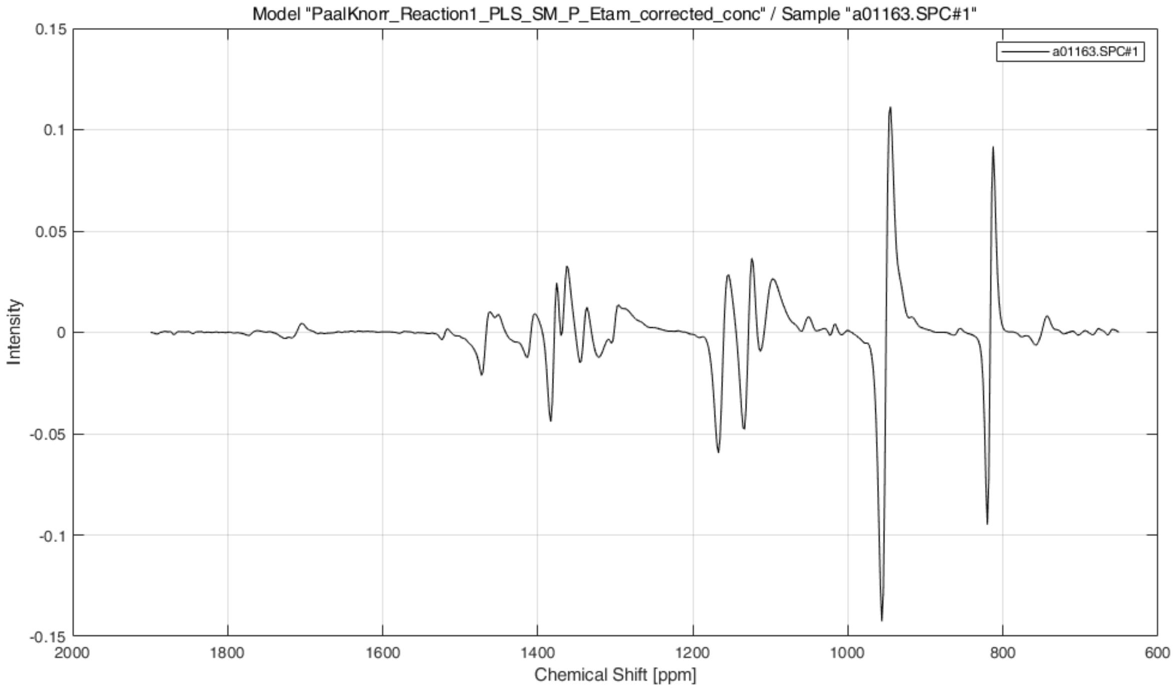

Figure 22: FTIR spectrum of the Paal Knorr reaction mixture forming one product after pretreatment.

Paal-Knorr reaction with two underlying reactions:

Training samples were obtained from calibration solutions from mixtures of 2,5-hexandione, ethylenediamine, product 1 and product 2 in concentrations ranging from 25-500 mmol/L including blank samples from toluene/methanol 2:1. Each sample was injected to the IR flow cell and at least five spectra were collected for each sample. The acquired training spectra were read into PEAXACT 5.3 (S-PACT) and processed with the following pretreatment conditions. The global range was set to 600-1900 cm^{-1}. A rubber band subtraction was used for baseline correction and the spectra were further processed using 1st derivative with a filter length of 5 for smoothing. A sample spectrum of the reaction mixture before and after pre-processing is shown in Figure 23 and Figure 24Fehler! Verweisquelle konnte nicht gefunden werden.. The following ranks were set for the components in the PLS model with the resulting root-mean-square error of calibration ($RMSE_C$) as a performance indicator: 2,5-hexandione (rank 3, R^2: 0.9964, $RMSE_C$: 7.36 mM, $RMSE_{CV}$: 7.85 mM), product 1 (rank 6, R^2: 0.9926, $RMSE_C$: 5.18 mM, $RMSE_{CV}$: 5.68 mM), product 2 (rank 5, R^2: 0.9969, $RMSE_C$: 3.45 mM, $RMSE_{CV}$: 3.72 mM). Product 1 and product 2 resulted in similar PLS loading. Therefore, distinguishing these was chemometrically challenging and errors in the mass balance of the experiments performed can be traced back to an error resulting from the PLS model.

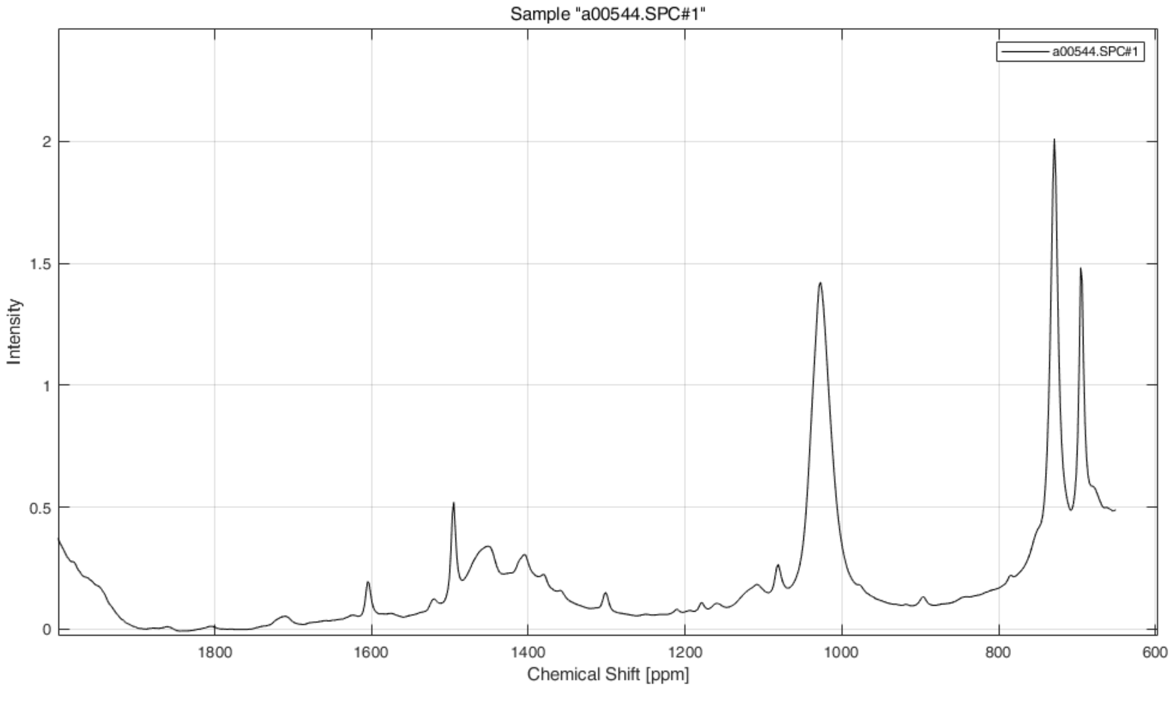

Figure 23: FTIR spectrum of the Paal Knorr reaction mixture forming two products before pretreatment.

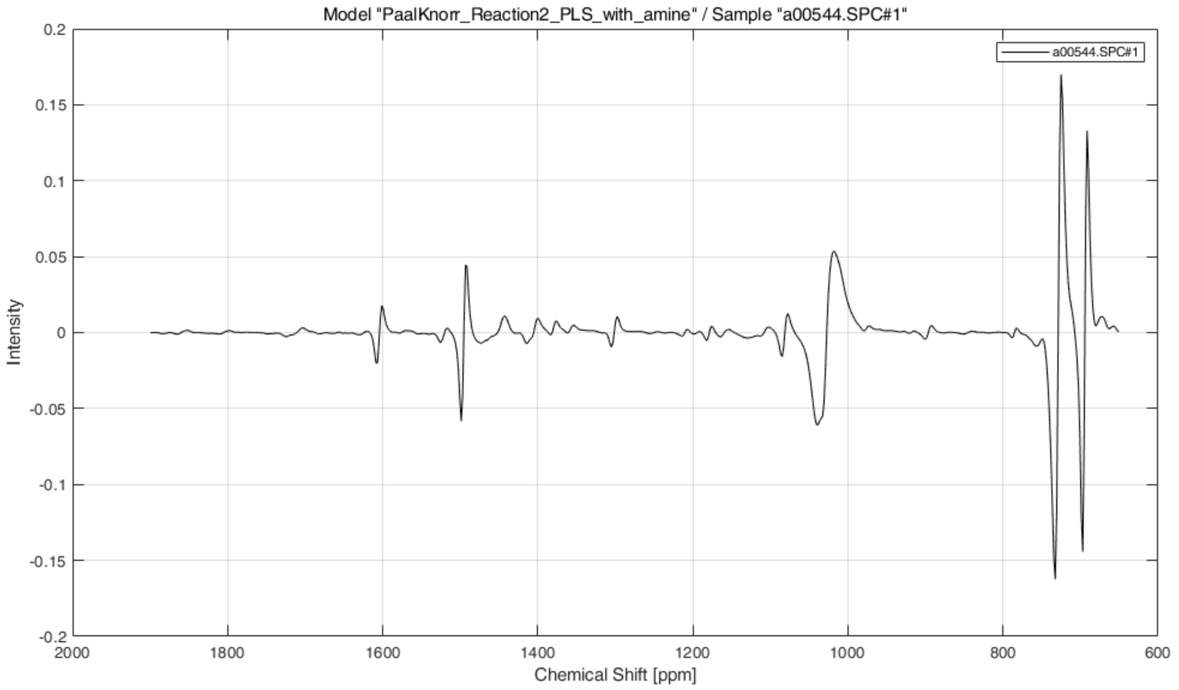

Figure 24: FTIR spectrum of the Paal Knorr reaction mixture forming two products after pretreatment.

COPASI was used as third-party software for initial kinetic fitting of the activation energy and Arrhenius factor simultaneously, starting from randomly generated initial values. [3]

Literaturverzeichnis

[1] „GitHub - FlowMate,“ [Online]. Available: https://github.com/SKenb/FlowMate/. [Zugriff am 01 2025].

[2] MathWorks, „MathWorks,“ [Online]. Available: https://de.mathworks.com/products/matlab.html. [Zugriff am 2 2025].

[3] S. Hoops, S. Sahle, R. Gauges, C. Lee, J. Pahle, N. Simus, M. Singhal, L. Xu, P. Mendes und U. Krummer, „COPASI—a COmplex PAthway SImulator,“ *Bioinformatics,* 10 2006.
